# TRP channel expression correlates with the epithelial–mesenchymal transition and high-risk endometrial carcinoma

**DOI:** 10.1007/s00018-021-04023-1

**Published:** 2021-12-22

**Authors:** Charlotte Van den Eynde, Katrien De Clercq, Rieta Van Bree, Katrien Luyten, Daniela Annibali, Frédéric Amant, Sileny Han, Els Van Nieuwenhuysen, Thaïs Baert, Karen Peeraer, Thomas Voets, Toon Van Gorp, Joris Vriens

**Affiliations:** 1 Laboratory of Endometrium, Endometriosis and Reproductive Medicine, Department of Development and Regeneration, KU Leuven, Herestraat 49 Box 611, 3000 Leuven, Belgium; 2 Laboratory of Ion Channel Research, Department of Cellular and Molecular Medicine, VIB Center for Brain and Disease Research, KU Leuven, Herestraat 49 Box 802, 3000 Leuven, Belgium; 3 Laboratory of Gynaecological Oncology, KU Leuven Herestraat 49, Box 7003, 3000 Leuven, Belgium; 4 Division of Gynaecological Oncology, Department of Gynaecology and Obstetrics, Leuven Cancer Institute, University Hospitals Leuven, Leuven, Belgium; 5 Leuven University Fertility Centre, University Hospital Leuven, Leuven, Belgium; 6 Centre for Gynecologic Oncology Amsterdam (CGOA), Antoni Van Leeuwenhoek-Netherlands Cancer Institute (AvL-NKI), University Medical Center (UMC), Amsterdam, The Netherlands

**Keywords:** TRP channels, Endometrium, Epithelial-to-mesenchymal transition (EMT), Mesenchymal-to-epithelial transmission (MET), Endometrial cancer

## Abstract

Transient receptor potential (TRP) channels excel in cellular sensing as they allow rapid ion influx across the plasma membrane in response to a variety of extracellular cues. Recently, a distinct TRP mRNA expression signature was observed in stromal cells (ESC) and epithelial cells (EEC) of the endometrium, a tissue in which cell phenotypic plasticity is essential for normal functioning. However, it is unknown whether TRP channel mRNA expression is subject to the phenotypic switching that occurs during epithelial to mesenchymal transition (EMT) and mesenchymal to epithelial transition (MET), and whether TRP channel mRNA expression is associated with aggressive phenotypes in endometrial cancer (EC). Here, we induced EMT and MET in vitro using in primary EEC and ESC, respectively, and analyzed expression and functionality of TRP channels using RT-qPCR and intracellular Ca^2+^ imaging. The outcome of these experiments showed a strong association between TRPV2 and TRPC1 mRNA expression and the mesenchymal phenotype, whereas TRPM4 mRNA expression correlated with the epithelial phenotype. In line herewith, increased TRPV2 and TRPC1 mRNA expression levels were observed in both primary and metastatic EC biopsies and in primary EC cells with a high EMT status, indicating an association with an aggressive tumor phenotype. Remarkably, TRPV2 mRNA expression in primary EC biopsies was associated with tumor invasiveness and cancer stage. In contrast, increased TRPM4 mRNA expression was observed in EC biopsies with a low EMT status and less aggressive tumor phenotypes. Taken together, this dataset proved for the first time that TRP channel mRNA expression is strongly linked to cellular phenotypes of the endometrium, and that phenotypic transitions caused by either experimental manipulation or malignancy could alter this expression in a predictable manner. These results implicate that TRP channels are viable biomarkers to identify high-risk EC, and potential targets for EC treatment.

**Supplementary Information:**

The online version contains supplementary material available at 10.1007/s00018-021-04023-1.

## Introduction

Cellular plasticity, defined as the ability of cells to reversibly assume different cellular phenotypes, is essential in several physiological processes like embryonic development and wound healing. The human endometrium comprises two major cell populations, the endometrial epithelial cells (EEC) and the underlying endometrial stromal cells (ESC), which possess remarkable cellular plasticity to facilitate monthly remodeling and regeneration. Indeed, both EEC and ESC alter their phenotype and behavior in a timely manner, allowing for the development of a receptive endometrium in response to fluctuations in reproductive hormones and growth factors. For instance, the luminal epithelial cells undergo an epithelial-to-mesenchymal transition (EMT) in response to an invading embryo, whereas a mesenchymal–epithelial transition (MET) is observed in ESC during decidualization [1, 2]. These intricate transitions are characterized by modifications in cellular morphology, physiology and function to meet the various requirements that facilitate pregnancy. However, dysregulation of these processes can result in uterine pathologies such as adenomyosis, endometriosis and endometrial carcinoma (EC).

During EC development, epithelial cells acquire a resilient and aggressive phenotype with mesenchymal features, which will facilitate malignant disease. As such, they gradually lose their polarity and cell–cell contacts and undergo dramatic remodeling of the cytoskeleton, thereby acquiring a migratory phenotype. This phenotypic switch is promoted by extracellular signals that are established by the tumor microenvironment, comprising immune cells, cancer-associated fibroblasts and the extracellular matrix. The constant dialogue between neoplastic cells and the tumor microenvironment can result in a never-ending loop of phenotypic alterations, ultimately resulting in metastatic disease [3]. Tumor metastasis in advanced stages of EC challenges current treatment methods and results in poor patient prognosis. Therefore, it is essential to gain novel insights into the interaction between cancer cells and their environment, and how this drives the metastatic cascade in EC.

Cellular sensitivity towards cues of the microenvironment is defined by the presence of specific receptors at the cell surface. Additionally, cells have a way to decode extracellular messages and transduce these signals into the desired response. Calcium ions (Ca^2+^) are perfect candidates to enable efficient signal transduction from receptor to effector. The steep gradient between cytosolic (low ~ 100 nM) and extracellular (high ~ 1.5 mM) Ca^2+^ concentrations allows for very specific intracellular signalization in response to the extracellular environment [4]. Indeed, extracellular Ca^2+^ entry, mostly mediated via Ca^2+^-permeable ion channels, has significant effects on the cellular phenotype. Therefore, the expression signature of Ca^2+^ permeable ion channels can be crucial for the cell, and modifications in this signature may result in abnormal cellular behavior [5–7].

Transient receptor potential (TRP) channels are (Ca^2+^ permeable) ion channels that act as cellular sensors as they are involved in the detection of temperature (heat and cold), chemical- and mechanical stimuli [8]. In mammals, 28 different TRP channel genes have been identified, divided into six subfamilies based on sequence homology: TRPA, TRPV, TRPC, TRPM, TRPP and TRPML [9]. Since TRP channel expression and functionality can be regulated by hormones and growth factors present in the endometrium, they are excellent candidates to translate and encode messages from the endometrial microenvironment into cellular responses [10]. Interestingly, earlier reports have identified a very distinct mRNA expression pattern of TRP channels in ESC and EEC [11–13] (Supplementary Fig. 1). This distinct TRP channel signature suggests a divergence in sensitivity and response of ESC and EEC towards the extracellular environment. As such, high mRNA expression of TRPV2 and TRPC1 was observed in ESC whereas high mRNA expression of TRPM4 characterized EEC. Divergent spatiotemporal calcium signals elicited by activation of these phenotype specific TRP channels might be involved in regulation of certain processes associated with the cell phenotype or facilitate the phenotypic transitions. Hence, alterations in this TRP channel expression pattern could have vast effects on their behavior, possibly facilitating malignant behavior. An overview of the current knowledge of TRP channel expression and activity in cellular phenotype and EMT is provided elsewhere [14]. In line herewith, modifications in TRP channel expression have been observed in several cancer types like prostate cancer, breast cancer and colon cancer [6, 15, 16]. However, the correlation between the mRNA expression of TRP channels in epithelial and mesenchymal cell phenotypes in healthy and malignant tissue has never been investigated.

In this study, we evaluated the relation between the TRP channel signature and the endometrial cell phenotype via in vitro induction of phenotypic transitions EMT and MET in healthy primary endometrial cells. Next, we aimed to assess the mRNA expression of TRP channels in EC tumors and in EC cell lines with variable EMT status and clinicopathological characteristics to evaluate the potential of TRP channel mRNA expression levels as predictors for high-risk EC. Here, we correlated for the first time the mRNA expression of TRP channels in EC with EMT status, and identified TRP channel signatures that can be used to predict high-risk EC.

## Material and methods

### Sample collection

Control endometrial samples were collected at the Leuven University Fertility Centre from patients of reproductive age who underwent diagnostic hysteroscopy during fertility treatment (S60959). Endometrial cancer biopsies were collected from patients undergoing hysterectomy at the Division of Gynecologic Oncology, UZ Gasthuisberg. All samples were obtained with the approval of the Ethical Committee of UZ Gasthuisberg, Leuven, Belgium (S62750, S60959, ML2524) and after signing of an informed consent form by the patient. Tumor histological subtype was determined using the WHO-classification. Tumor stage and grade were determined via FIGO-classification.

### Primary endometrial cell cultures

#### Healthy EEC and ESC

The protocol used to establish primary endometrial stromal cell (ESC) and endometrial epithelial cell (EEC) cultures was described earlier [11, 13]. Briefly, healthy human endometrial biopsies were manually minced into small pieces and incubated with 0.2% collagenase type IA (Sigma-Aldrich) in phenol-red free low glucose Dulbecco’s modified Eagle’s Medium (DMEM)/F-12 (Gibco, Invitrogen) for either 60 min at 37 °C while shaking or overnight at 4 °C. ESC were collected by pouring the digestion mix trough a 20 µm cell strainer (Pluriselect) and centrifuging the supernatant at 2000 rpm. ESCs were resuspended and seeded in ESC growth medium containing DMEM/F12 supplemented with 10% fetal bovine serum (FBS), 0.2% gentamycin and 0.2% amphotericin B. The epithelial fraction was collected by backwashing the cell strainer with EEC growth medium consisting of phenol-red free, low glucose DMEM, 10% FBS, 20% MCDB-105, 5 µg/ml insulin, 0.2% gentamycin and 0.2% amphotericin B. The flow-through was mechanically disrupted by gently pipetting the solution up and down, collected in a T75 flask and incubated for a minimum time period of 30 min at 37 °C in 5% CO_2_. After incubation, non-attached cells were collected and centrifuged at 1500 rpm, resuspended in EEC growth medium and seeded in the appropriate cell culture plates for further experiments. Cells were kept at 37 °C in 5% CO_2_ and the medium was replaced every 2 days.

#### Endometrial cancer cells

Primary endometrial cancer cell cultures (EM018a, EM033, EM012 and EM046) were kindly provided by one of authors (FA). The isolation of cancer cells from biopsies and culturing conditions are described elsewhere [17]. Low passage (p3) cells were collected for RT-qPCR analysis when they reached confluence.

### Cell treatments

#### Decidualization

After reaching 80–90% cell confluence, primary ESC were treated with 0.5 mM 8-bromoadenosine cyclic monophosphate sodium (8-Br-cAMP) (Sigma-Aldrich) and 1 μM medroxyprogesterone acetate (MPA) (Sigma-Aldrich) for 5 days in medium containing 2% FBS [18]. Successful decidualization was validated via upregulation of PRL mRNA expression.

#### EMT

Primary EEC were treated with 1X StemXVivo EMT inducing medium supplement (R&D Systems) for 5 days in medium containing 2% FBS. EMT inducing medium was renewed every 2 days. Successful EMT was validated via upregulation and downregulation of mesenchymal and epithelial marker gene mRNA expression.

### RT-qPCR

#### RNA extraction and cDNA synthesis

*Cell cultures* Total RNA was isolated using the RNeasy Mini kit (Qiagen), according to the manufacturers’ guidelines. RNA concentration was assessed by use of the Nanodrop (Isogen Life Science). cDNA was generated from 1 μg of RNA using the First-strand cDNA Synthesis Kit (Thermofisher Scientific).

*Tissues* Tissue was homogenized by the use of a power homogenizer (Polytron) and total RNA was extracted with TriPure Isolation Reagent (Roche). RNA concentrations were assessed with the Nanodrop (Isogen Life Science) and RNA quality was assessed using an Experion RNA StdSens Analysing kit (Bio-Rad). 1 µg RNA was subsequently used for cDNA synthesis using the High-Capacity cDNA Reverse Transcription Kit (Life Technologies).

#### RT-qPCR

RT-qPCR was performed on triplicate cDNA samples using specific TaqMan gene expression assays in the StepOne PCR system. Hypoxanthine Phosphoribosyltransferase 1 (HPRT1) and Phosphoglycerate Kinase 1 (PGK1) were used as endogenous controls in cells. HPRT1, PGK1, actin beta (ACTB) and glyceraldehyde 3-phosphate dehydrogenase (GAPDH) were used as endogenous controls in EC cancer biopsies tissue.

### Functional measurements

#### Pharmacology

TRPV2 activity was assessed by the application of 50 µM Δ^9^*-*tetrahydrocannabinol (THC), kindly provided by prof G. Appendino and prof F. Pollastro [19], in the presence of the cannabinoid receptor 1 and 2 (CB1 and 2) blockers AM251, AM630 and GPR55 blocker ML193 (all at a dose of 1 µM) (Sigma-Aldrich). Ionomycin (2 µM, Sigma-Aldrich) was applied at the end of every experiment as a positive control. 

#### Calcium microfluorimetry

Intracellular Ca^2+^ measurements were performed as previously described [20]. Cells were incubated with 2 µM Fura-2 acetoxymethyl ester (Biotium) for 30 min at 37 °C. Fluorescent signals were evoked during alternating illumination at 340 and 380 nm using a Lambda XL illuminator (Sutter instruments) and recorded using an Orca Flash 4.0 camera (Hamamatsu Photonics Belgium) on a Nikon Eclipse Ti fluorescence microscope (Nikon). The imaging data were recorded and analyzed using NIS-elements software (Nikon). Absolute calcium concentrations were calculated from the ratio of the fluorescence signals at both wavelengths (F340/F380) after correction for the individual background fluorescence signals, using the Grynkiewicz equation. The standard solution contained (in mM) 150 NaCl, 2 CaCl_2_, 1 MgCl_2_, 10 d-glucose and 10 4-(2-hydroxyethyl)-1-piperazineethanesulfonic acid (HEPES) (pH 7.4 was adjusted with NaOH). For all measurements, cells were considered responders if the calcium influx during agonist application exceeded 50 nM and when the highest value of the derivative of the calcium trace during the application of an activator exceeded at least 3 times the standard deviation of the derivative during basal conditions. Calcium amplitudes were calculated as the difference between the maximum calcium concentration and the basal calcium level of responding cells during the application of an activator as described elsewhere [11].

### Immunohistochemistry

Primary EEC and EC cells (EM018a, EM033, EM012 and EM046) were seeded in a 12-well plate on collagen-coated (Sigma-Aldrich) coverslips (Karl Hecht). After EMT induction, cells were fixed with 4% formaldehyde for 10 min, permeabilized with 0.2% Triton X-100 for 10 min and blocked with 5% goat serum for 2 h. The primary antibodies were incubated overnight at 4 °C. The following primary antibodies were used: mouse monoclonal anti-human E-cadherin (1/30, Abcam), rabbit monoclonal anti-human vimentin (1/100, Abcam), mouse monoclonal anti-human cytokeratin (1/100 Agilent), mouse monoclonal anti-human MMP2 (1/100, Abcam), and mouse monoclonal anti-human α-smooth muscle actin (1/100, Agilent). The secondary antibodies (1:1000 in 0.5% goat serum, AlexaFluor488-conjugated anti-mouse IgG and anti-rabbit, AlexaFluor647-conjugated anti-mouse and anti-rabbit) were applied for 1 h at room temperature. Triple washing with PBS was performed between each step. Finally, the coverslips were mounted in medium containing 4′,6-diamidino-2-phenylindole (DAPI) (Vectashield, Vector Laboratories). Images were taken using the Nikon Fluorescence microscope (CIE i) taking care to use the same exposure and gain settings for each type of staining to compare fluorescence intensity.

### Protein expression analysis via western blot (WB)

Cell collection and western blot for protein expression analysis are described elsewhere [21]. Briefly, vehicle-treated and decidualized cells were collected in the whole cell lysis buffer containing (in mM) 50 HEPES, 150 NaCl, 1.5 MgCl_2_, 1 EDTA, 1 PMSF, 10% glycerol, 1% Triton X-100, supplemented with protease inhibitor cocktail (Sigma-Aldrich). Next, whole cell lysate was prepared for SDS-page by adding fourfold concentrated Laemmli sample buffer (Biorad) substituted with 2-β-mercaptoethanol (99%, Sigma-Aldrich) and heating to 95° for 5 min. Samples were evaluated by SDS-PAGE using NuPAGE Novex Bis–Tris 4–12% Gels (Life Technologies) according to the manufacturer’s protocol. Separated proteins were transferred to a PVDF membrane (Millipore) and immersed for 1 h in blocking solution (5% w/v nonfat dry milk in TBS containing 0.1% Tween-20). The membranes were probed with anti-hTRPV2 (in-house, kindly provided by Prof. V. Flockerzi (Universität des Saarlandes, Homburg), 1/100), GAPDH (Sigma-Aldrich, 1/2000) and IGFBP-1 (Thermofisher Scientific, 1/2000) antibodies overnight at 4 °C. Next, the membranes were washed in TBST and incubated with horseradish peroxidase (HRP)-conjugated secondary antibodies (1/5000; Cell Signaling Technology) for 1 h at room temperature. Immunoreactive complexes were visualized using ECL Western blotting detection reagent (GE Healthcare) and ChemiDoc MP Imaging System (version 5.01 Beta, Bio-rad Laboratories).

### Data analysis

Ca^2+^ microfluorimetric data were analyzed using home-written routines in IgorPro 6.37 (WaveMetrics), and GraphPad Prism version 9 (GraphPad Software) was further used for data display. Statistical analysis was conducted via GraphPad Prism and SPSS statistics 27. Multiple imputation was performed to account for missing values in clinical data. The relationship between clinical pathologic features and the expression of TRP channels in EC was analyzed using linear regression methods. Correlations between TRP channels and markers were calculated using Pearson’s correlation. To assess whether TRP channel expression could be a valuable predictor for EC relapse, logistic regression methods were used. All statistics on qRT-PCR data were performed on dCT values. Normality of the data was assessed via Kolmogorov–Smirnov test (> 50 samples) or Shapiro–Wilk test (< 50 samples). Specific statistical tests were stated in figure legend. Results were considered significant when *p* < 0.05.

## Results

### Cell phenotypic switching alters expression of TRP channels in healthy cells

**Fig. 1:**
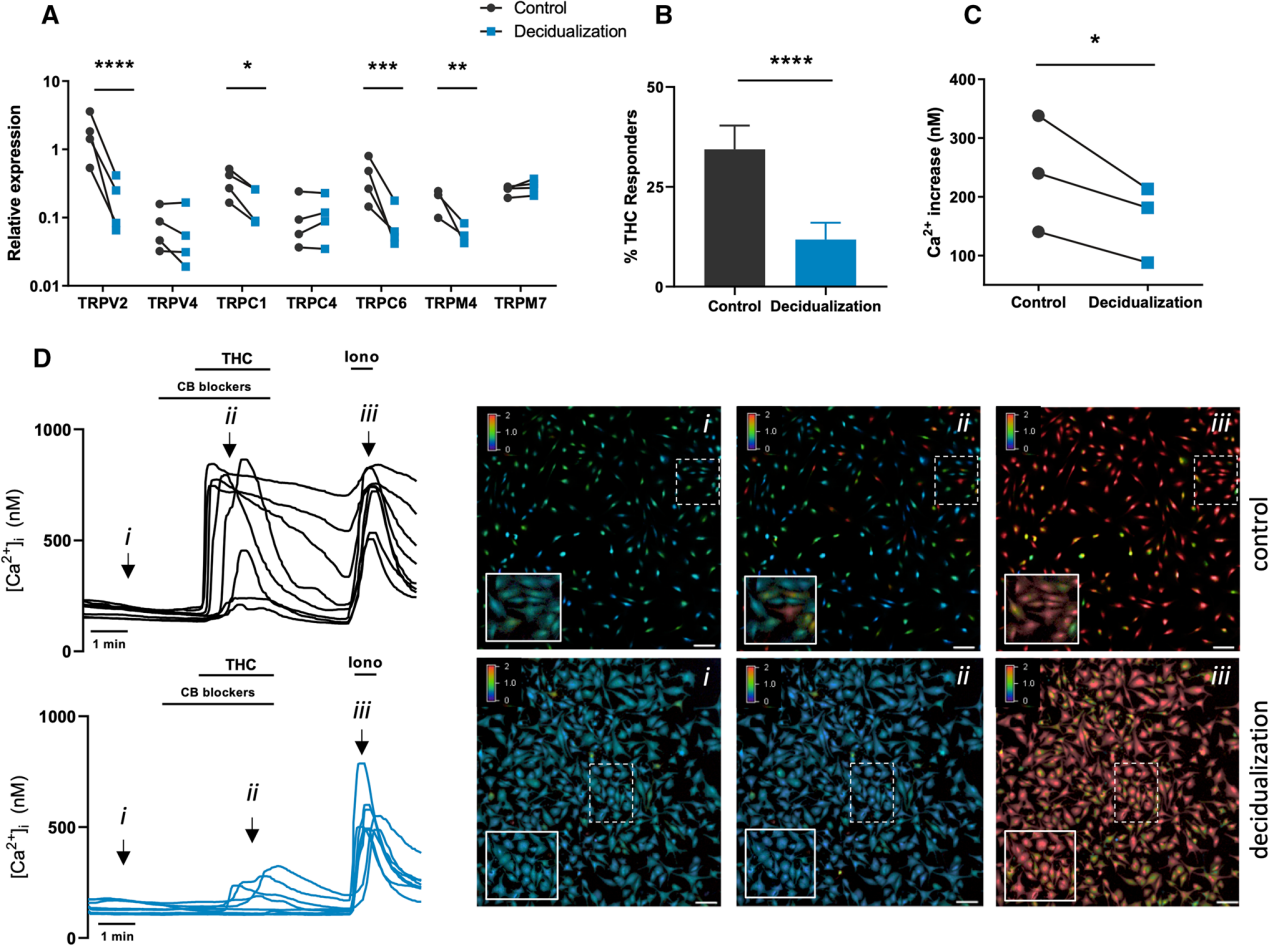
TRP channels in decidualization. **A** Quantitative RT-qPCR showing TRP channel mRNA expression (TRPV2, TRPV4, TRPC1, TRPC4, TRPC6, TRPM4 and TRPM7) in control and decidualized ESC. Results are shown as relative expression to the geometric mean of housekeeping genes HPRT and PGK. *p < 0.05, **p < 0.01, ***p < 0.001, ****p < 0.0001 using 2-way ANOVA and Sidak’s multiple comparison test on ΔCT values. *N* = 4 patient samples (p.s). **B** Percentage (%) of THC (50 µM) responders in control (*n* = 2356) vs decidualized (*n* = 3808) ESC. ****p < 0.01 using Fisher’s exact test. *N* = 3 p.s, *n* = 19–23 coverslips per condition. Data are represented as mean ± SEM. **C** Mean Ca^2+^ amplitude of all cells during THC application, represented as the difference between the peak value and the baseline value. *p < 0.05 using Mann–Whitney test. *N* = 3 p.s. *n* = 19–23 coverslips per condition. **D** Representative Ca^2+^ traces of THC-induced intracellular calcium changes ([Ca^2+^]_i_) in control vs. decidualized ESC with each line representing a cell. (i) Representative colour-coded Fura-2 [Ca^2+^]_I_ ratio images during measurement of ESC indicated in graph (**D)**:basal situation (i), after THC application (ii), and after application of ionomycin (iii). Pseudo-colour ratio images were obtained using Nikon software. Scale bar = 100 µm

Decidualization of ESCs entails drastic remodeling of the fibroblast phenotype into epithelioid-like secretory cells. Decidual cells secrete several factors important for embryonic growth, such as prolactin (PRL) and insulin-like growth factor-binding protein 1 (IGFBP-1). To induce the MET-like decidualization process, healthy primary ESCs were supplemented with 8-Br-cAMP and MPA for an incubation period of 5 days. Afterwards, the decidualization of ESC was verified by the significant upregulation of PRL mRNA expression compared to vehicle stimulated ESCs (Supplementary Fig. 2A). Next, the mRNA expression pattern of several TRP channels was investigated in control and decidualized ESC. We only assessed expression of TRP channels that were shown in previous work to be expressed in the endometrium, namely TRPV2, TRPV4, TRPC1, TRPC4, TRPC6, TRPM4 and TRPM7 (Supplementary Fig. 1). These results showed a significant downregulation of the mRNA expression levels of TRPV2, TRPC1, TRPC6 and TRPM4 after decidualization, whereas expression levels of TRPV4, TRPC4, and TRPM7 were unaltered (Fig. 1A). TRPV2 was most abundantly expressed in ESC and underwent the most pronounced reduction in expression levels during decidualization (± tenfold). Interestingly, the reduced levels of TRPV2 mRNA were both time- and 8-Br-cAMP dose dependent (Supplementary Fig. 2B and C), underlining a strong association with the fibroblast phenotype. Next, the functional expression of TRPV2 was investigated using calcium microfluorimetry via a standard protocol [11, 20, 22]. Stimulation of primary ESCs by the TRPV2 agonist, THC (50 µM) with co-application of CB receptor blockers, induced a robust calcium influx (Δ[Ca^2+^]_i_ = 239.5 ± 56.9 nM (mean ± SEM)) in 34.4 ± 6.0% of the cells. In contrast, application of THC in decidualized ESC resulted in significantly diminished calcium influx ((Δ[Ca^2+^]_i_ = 161 ± 37.61 nM) and a lower number of responding cells (~ 11.8 ± 4.2%) (Fig. 1B–D). Finally, decreased TRPV2 protein expression after decidualization was validated on western blot (Supplementary Fig. 2D). These findings suggest that during MET-related processes like decidualization, the TRP channel signature undergoes alterations at mRNA level and, in the case of TRPV2, also at functional level.

**Fig. 2:**
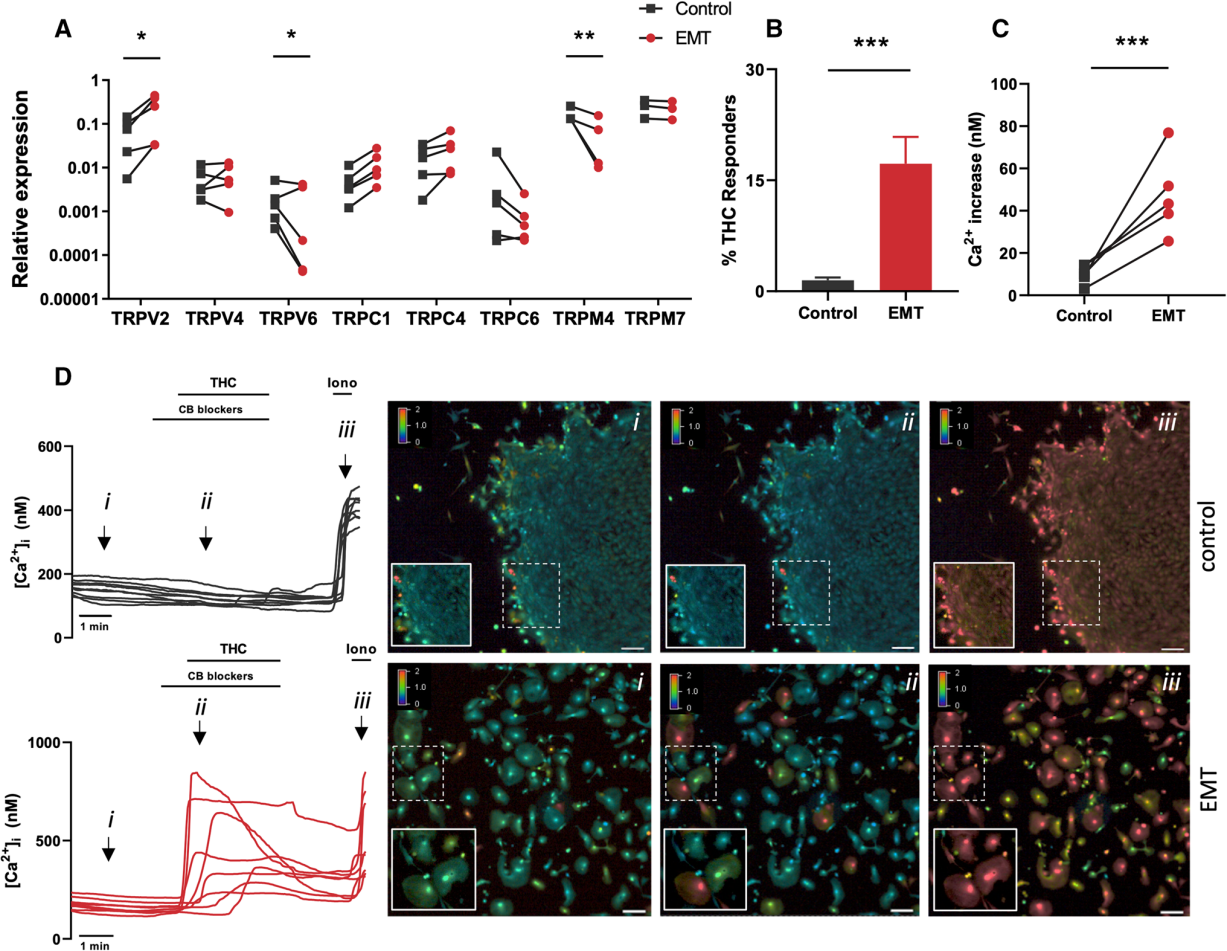
TRP channels in EMT: **A** Quantitative RT-qPCR showing TRP channel mRNA expression (TRPV2, TRPV4, TRPV6, TRPC1, TRPC4, TRPC6, TRPM4 and TRPM7) in control and EMT EEC. Results are shown as relative expression to the geometric mean of housekeeping genes HPRT and PGK. *p < 0.05, **p < 0.01 using two-way ANOVA and Sidak’s multiple comparison test on ΔCT values. *N* = 3–5 p.s. **B** Percentage (%) of THC responders in control (*n* = 1744) vs EMT (*n* = 1765) EEC. ***p < 0.001 using Fisher’s exact test. *N* = 5 p.s, *n* = 14 measurements. Data are represented as mean ± SEM. **C** Ca^2+^ amplitude in all cells during THC application, represented as the difference between the peak value and the baseline value. *p < 0.05 using Mann–Whitney test. *N* = 5 p.s, *n* = 14 measurements. **D** Representative Ca^2+^ traces of THC-induced [Ca^2+^]_i_ changes in control vs. EMT. Each line represents a single EEC. (i) Representative colour-coded Fura-2 [Ca^2+^]_I_ ratio images during measurement of EEC indicated in graph **D**: basal situation (i), after THC application (ii), and after application of ionomycin (iii). Pseudo-colour ratio images were obtained using Nikon software. Scale bar = 100 µm

Next, the TRP channel expression was investigated in EEC undergoing EMT. To induce EMT, EECs were incubated EMT-inducing medium supplement for 5 days. Successful EMT was verified by increased expression of mesenchymal markers MMP2, MMP9, ZEB1, ACTA, CDH2 and VIM, in combination with decreased expression of epithelial markers CDH1, KRT18, EPCAM and MMP7 (Supplementary Fig. 3). Interestingly, inducing EMT in EEC was associated with significant upregulation of TRPV2 mRNA levels (fourfold), whereas expression levels of TRPM4 and TRPV6 were significantly downregulated. The expression levels of TRPV4 and TRPM7 were unaltered during the cell phenotype switch (Fig. 2A). The increased mRNA expression of TRPV2 was further validated at the functional level via calcium imaging experiments. These experiments showed the significantly enhanced TRPV2 functionality, demonstrated by increased percentage of THC-responding cells compared to vehicle treated cells (17.2 ± 3.6% vs. 1.5 ± 0.4%, respectively) and an increased calcium amplitude (Fig. 2B–D). Taken together, these results suggest a cell phenotypic TRP channel expression signature, where the mesenchymal phenotype is characterized by increased mRNA expression of TRPV2, TRPC1, TRPC4, while the epithelial phenotype is characterized by a pronounced mRNA expression of TRPM4 and TRPV6.

### TRP channel expression in primary and metastatic endometrial cancer positively correlates with mesenchymal marker gene expression

Phenotypic switching is a malignant hallmark of endometrial cancer pathophysiology. EEC acquire mesenchymal characteristics, resulting in a more invasive cell phenotype, which, together with tumor microenvironment, contributes to disease progression and dissemination. Given the strong association between cell phenotype and the typical TRP channel expression, the representation of TRP channel mRNA expression in both primary and metastatic EC biopsies was investigated.

**Fig. 3:**
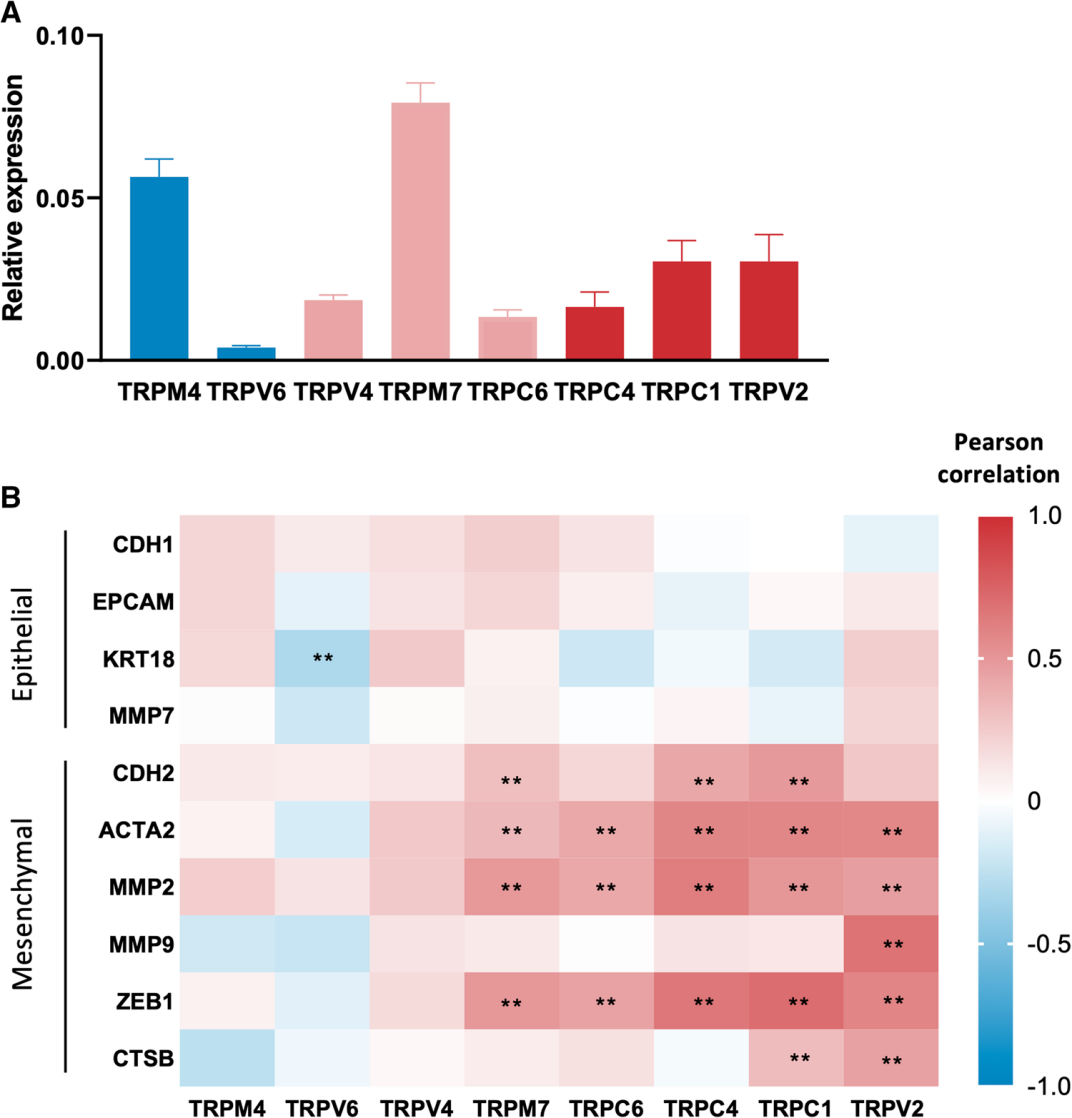
TRP channels in primary EC biopsies: **A** Quantitative RT-qPCR showing TRP channel mRNA expression (TRPV2, TRPV4, TRPV6, TRPC1, TRPC4, TRPC6, TRPM4 and TRPM7) in EC cancer biopsies. Results are shown as relative expression to the geometric mean of housekeeping genes HPRT, PGK, ACTB and GAPDH. Colors indicate association with the epithelial phenotype (blue), the mesenchymal phenotype (red) or both (pink) in healthy endometrial cells. Data are represented as mean ± SEM. *N* = 54 p.s. **B** Pearson correlation coefficients between TRP channels and epithelial (CDH1, EPCAM, KRT18, MMP7) and mesenchymal (CDH2, ACTA2, MMP2, MMP9, ZEB1 and CTSB) marker genes in primary EC biopsies. Red indicates a positive correlation, while blue indicates a negative correlation. **p < 0.01. *N* = 54 p.s

First, the mRNA expression of TRP channels, as well as of mesenchymal and epithelial markers was assessed in 54 patient biopsies collected from the primary tumor site via RT-qPCR. High expression of TRPM4 and TRPM7 was detected in primary tumor biopsies, while TRPV4, TRPC4, TRPC6 and especially TRPV6 expression was limited, while the expression of TRPC1 and TRPV2 are moderately expressed (Fig. 3A). Next, correlations between the mRNA expression levels of the different TRP channels and mesenchymal and epithelial markers was investigated. Expression of TRPV2, TRPC1, TRPC4, TRPC6 and TRPM7 significantly correlated with mesenchymal markers (Fig. 3B). Interestingly, multiple linear regression analysis identified that the gene expression of TRPV2, TRPC1, TRPC4, TRPC6 and TRPM7 was significantly related to the mesenchymal marker genes present in the model. Notably, expression levels of TRPV2 and TRPC1 were most strongly correlated with mesenchymal marker genes (*R*^2^ = 0.703 and *R*^2^ = 0.713, respectively) and were further considered to identify significant contributors to predict their expression (Supplementary Table 1). Interestingly, MMP2 and MMP9 expression were significant contributors in the model for predicting TRPV2 expression, whereas MMP7, CTSB, MMP9 and ZEB1 were significantly contributing to predict TRPC1 expression (Supplementary Table 2).

**Fig. 4:**
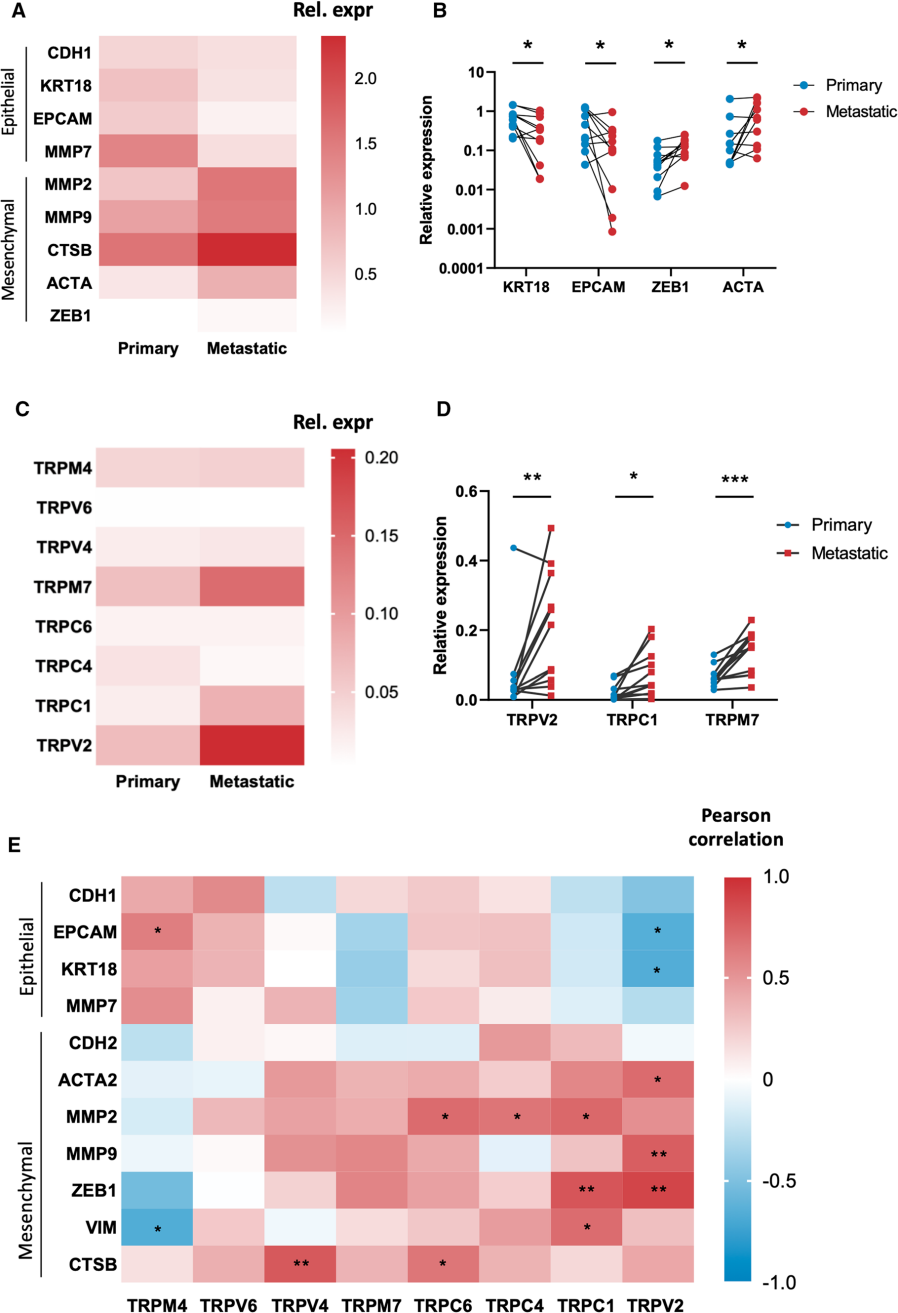
TRP channels in metastatic EC biopsies: **A**, ** C** Heat maps of mRNA expression of epithelial (CDH1, KRT18, EPCAM, MMP7) and mesenchymal (MMP2, MMP9, CTSB, ACTA2) markers and TRP channels (TRPV2, TRPV4, TRPV6, TRPC1, TRPC4, TRPC6, TRPM4 and TRPM7) in primary and metastatic tumor biopsies. *N* = 22 p.s. Results are shown as mean relative expression to the geometric mean of housekeeping genes HPRT, PGK, ACTB and GAPDH. **B**, ** D** Relative expression of marker genes and TRP channels displaying a significant association with either the primary or metastatic nature of the tumour biopsy. *p < 0.05 **p < 0.01, ***p < 0.001 using univariate linear regression with mRNA expression (ΔCT) as dependent variable and biopsy status as independent variable. *N* = 22 p.s. **E** Pearson correlation coefficients between TRP channels and epithelial (CDH1, EPCAM, KRT18, MMP7) and mesenchymal (CDH2, ACTA2, MMP2, MMP9, ZEB1 and CTSB) marker genes in metastatic EC biopsies. Red indicates a positive correlation, while blue indicates a negative correlation. *p < 0.05 **p < 0.01. *N* = 22 p.s

Next, we aimed to evaluate the mRNA expression of TRP channels and marker genes in tumor biopsies obtained from metastatic sites compared to primary tumor biopsies. Metastatic biopsies were acquired from 11 of the 54 patients analyzed above, allowing direct comparison of gene expression between primary and metastatic tumors from the same patient. As expected, metastatic biopsies exhibited significantly decreased expression of epithelial marker genes and showed an increased expression of typical mesenchymal genes, suggesting an increased EMT status (Fig. 4A). Univariate linear regression revealed a significant negative association of KRT18 and EPCAM expression, and a significant positive association of ZEB1 and ACTA2 expression with metastatic biopsies (Fig. 4B). Interestingly, expression levels of TRPV2, TRPC1 and TRPM7 were significantly increased in the metastatic biopsy compared to the primary biopsy (Fig. 4C, D).

Similar, but more pronounced, correlation patterns were observed in metastatic biopsies (Fig. 4E). As such, TRPV2 and TRPC1 expression was positively correlated with mesenchymal marker gene expression and negatively correlated with epithelial marker gene expression. Contrary, TRPM4 expression correlated positively with epithelial marker genes and negatively with mesenchymal marker genes. In accordance with the results obtained in primary biopsies, a high correlation for TRPV2 and TRPC1 with mesenchymal gene expression was observed (*R*^2^ = 0.867 and *R*^2^ = 0.728, respectively) (Supplementary Table 3). In metastatic biopsies, ZEB1 was the significant predictor for both TRPV2 and TRPC1 in the regression model (Supplementary Table 4).

### Relationship between TRP channel expression and clinical parameters

**Fig. 5:**
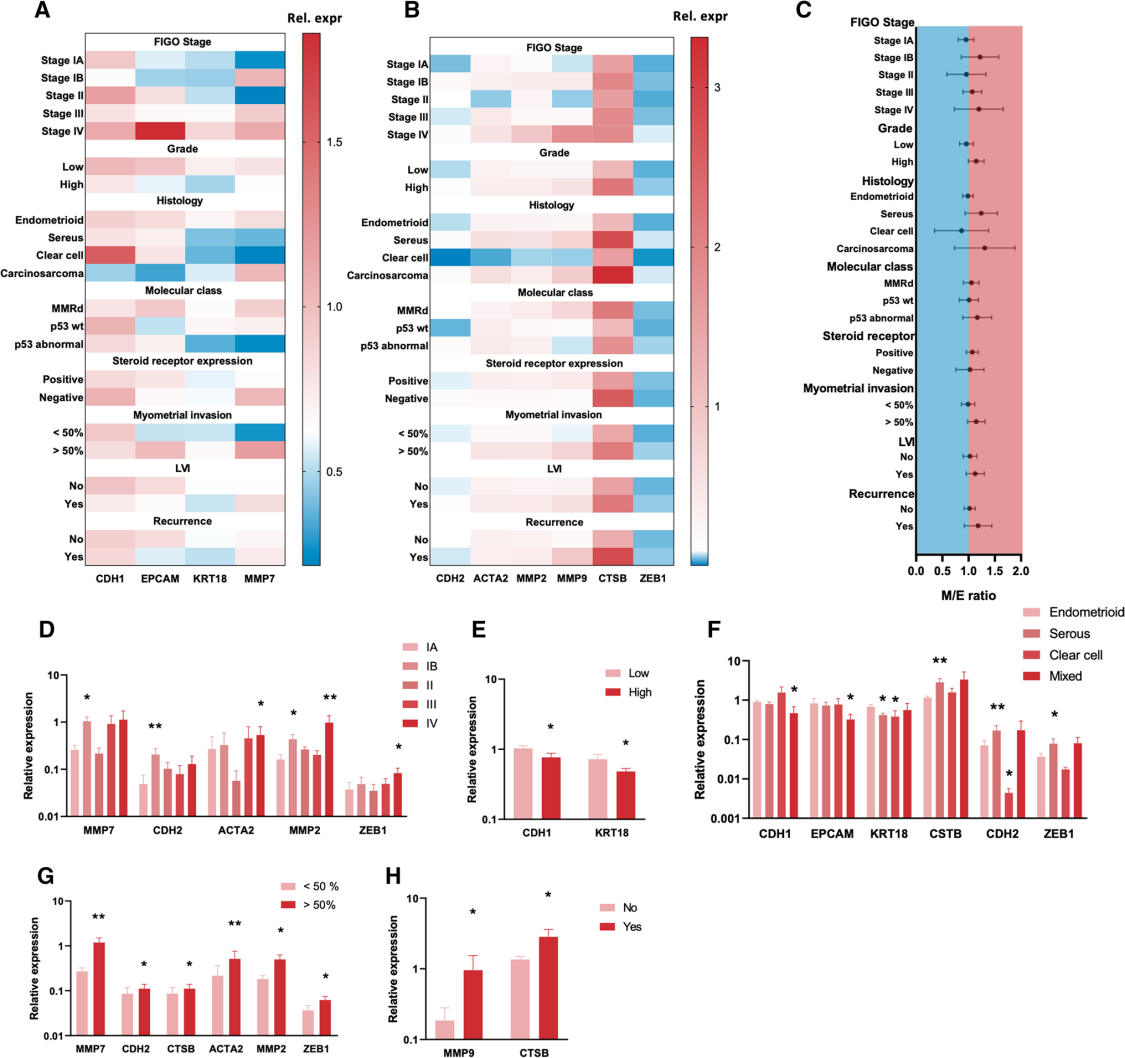
Relationship between marker gene expression and clinical parameters: **A**,**  B** Heat maps of mRNA expression of epithelial (CDH1, KRT18, EPCAM, MMP7) and mesenchymal (MMP2, MMP9, CTSB, ACTA2) markers between different clinical parameters (FIGO stage, grade, histology, molecular class, steroid receptor expression, myometrial invasion, LVI and recurrence) in primary EC biopsies. Results are shown as mean relative expression to the geometric mean of housekeeping genes HPRT, PGK, ACTB and GAPDH. *N* = 54 p.s. **C** Mesenchymal/epithelial ratio between different clinical parameters shown as mean ± 95% CI. Colours indicate ratio’s < 1 (blue) and > 1 (red). The ratio was constructed for each biopsy using median expression values. **D**–**H** Relative expression of marker genes displaying significant associations with certain clinical parameters: stage (compared to Ia) (**D**), grade (**E**), histology (compared to endometrioid) (**F**), myometrial invasion (**G**) and recurrence (**H**). Data are represented as mean ± SEM. *p < 0.05 **p < 0.01 using univariate linear regression with mRNA expression (ΔCT) as dependent variable and clinical parameters as independent variable. *N* = 54 p.s

Next, the potential correlation between the variations in TRP channel mRNA levels and typical marker genes was assessed to explain the pathophysiological characteristics of the tumor biopsy. First, expression of epithelial and mesenchymal marker genes was assessed between several clinical parameters, including FIGO stage, grade, tumor histology, molecular classification, steroid dependency, presence of myometrial and lymph vascular invasion (LVI), and disease recurrence (Fig. 5A, B). Patient statistics are presented in Supplementary Fig. 4. Median expression values were used to construct a mesenchymal/epithelial (M/E) ratio for each biopsy, a value indicative for high-risk EC and (Fig. 5C). Despite the large overlap in the 95% CI intervals between the groups, a trend towards and increased M/E ratio was observed in late stage (from IB onwards) and high-grade tumors, as well as in tumors with serous and carcinosarcoma histology and p53 abnormal molecular classification (abnormal p53 expression, high risk). Furthermore, an increased M/E ratio was observed in tumors expressing steroid receptors, tumors that invade up to 50% of the myometrium and displayed LVI. Finally, patients who experienced recurrence showed an elevated M/E ratio in the primary tumor biopsy.

Subsequently, univariate linear regression was used to identify significant associations between epithelial and mesenchymal marker genes and clinical parameters (Fig. 5D–H). Late-stage tumors showed increased expression of CDH2, ACTA2, MMP2 and ZEB1 compared to stage IA tumors (Fig. 5D). Reduced expression of CDH1 and KRT18 was significantly associated with high-grade tumors and non-endometrioid tumor histology, while increased expression CTSB, CDH2 and ZEB1 could be observed in serous tumors (Fig. 5E, F). No significant associations were observed between marker genes and molecular subclasses, nor when comparing steroid receptor positive and negative tumors and presence of LVI. Increased expression of CDH2, CTSB, ACTA2, MMP2 and ZEB1 was associated with increased myometrial invasion, and expression of CTSB and MMP9 was significantly associated with tumor recurrence (Fig. 5G, H). These results suggest that high-risk EC tumors display an increased EMT status.

**Fig. 6:**
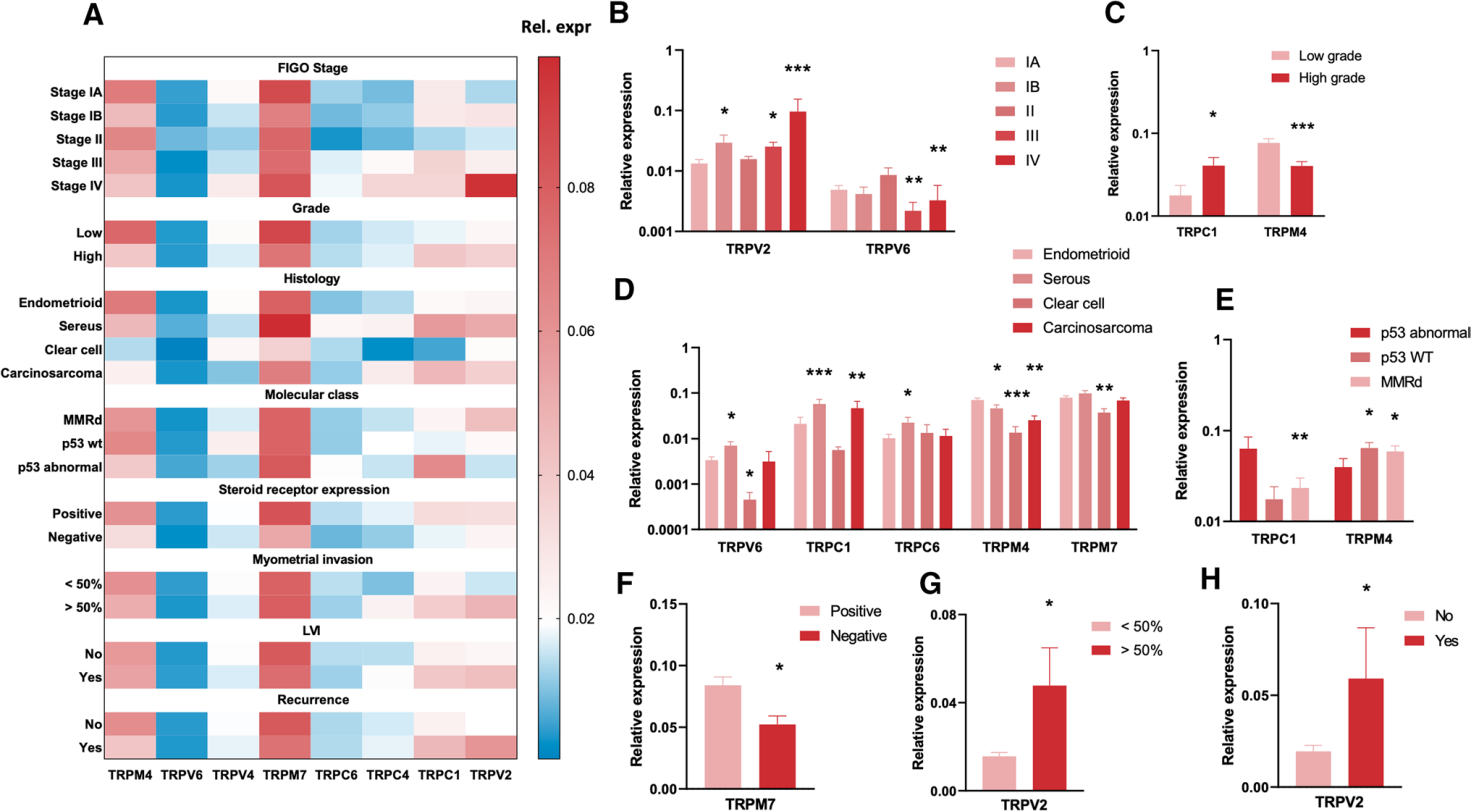
Relationship between TRP channel expression and clinical parameters: **A** Heat map of mRNA expression of TRP channels (TRPV2, TRPV4, TRPV6, TRPC1, TRPC4, TRPC6, TRPM4 and TRPM7) between different clinical parameters (FIGO stage, grade, histology, molecular class, steroid receptor expression, myometrial invasion, LVI and recurrence) in primary EC biopsies. Results are shown as mean relative expression to the geometric mean of housekeeping genes HPRT, PGK, ACTB and GAPDH. *N* = 54 p.s. **B**–**H** Relative expression of TRP channels displaying significant associations with certain clinical parameters: stage (compared to IA) (**B**), grade (**C**), histology (compared to endometrioid) (**D**), molecular subclass (compared to p53 abnormal) (**E**), steroid receptor expression (**F**), myometrial invasion (**G**) and recurrence (**H**). Data are represented as mean ± SEM. *p < 0.05 **p < 0.01 using univariate linear regression with mRNA expression (ΔCT) as dependent variable and clinical parameters as independent variable. *N* = 54 p.s

Interestingly, evaluating TRP channel mRNA expression signatures across clinical parameters reveals that TRPV2 and TRPC1 expression is associated with high-risk EC biopsies characterized by a high EMT status, while TRPM4 expression is associated with low-risk EC biopsies characterized by a low EMT status (Fig. 6A). Indeed, increased TRPV2 mRNA expression was significantly associated with stage IB, stage III and stage IV tumors compared to stage IA tumors (Fig. 6B), deep myometrial invasion (Fig. 6G) and recurrence (Fig. 6H). Expression of TRPC1 was significantly associated with high-grade tumors (Fig. 6C), tumors with serous and carcinosarcoma histology (Fig. 6D) and p53 abnormal (abnormal p53 expression, high risk) classified tumors (Fig. 6E). In contrast, high TRPM4 expression was significantly associated with low-grade tumors (Fig. 6C), endometrioid histology (Fig. 6D) and lower risk p53 WT (wild type p53 expression) and MMRd tumors (mismatch repair deficient) (Fig. 6E).

### TRPV2 mRNA expression in primary biopsies as predictor for recurrence

**Fig. 7:**
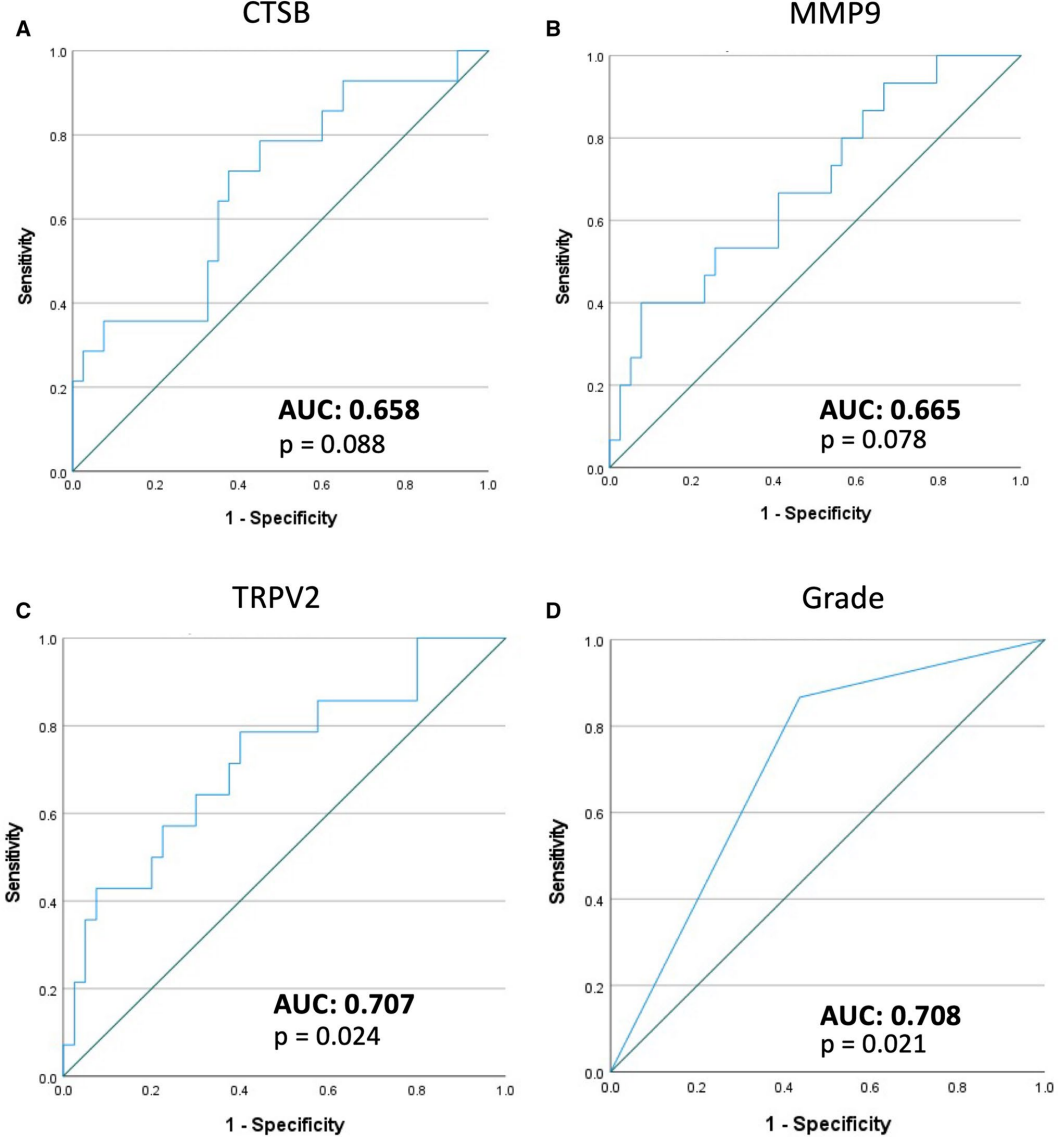
TRPV2 as predictor for disease recurrence. **A**–**D** Receiver operating characteristic (ROC) curves for genes (**A**: CTSB, **B**: MMP9, **C**: TRPV2) and clinical parameters (**D**: grade) that showed a significant association with disease recurrence. Area under the curve (AUC) and *p*-values are indicated on the panels

Interestingly, previous results showed that recurrence of the disease was significant associated with increased levels of CTSB and MMP9, and TRPV2 expression (Figs. 5H and 6H). Thus, univariate logistic regression with recurrence as dependent variable and gene expression as independent variable was used to assess whether these genes in primary tumors would be able to predict recurrence. Remarkably, CTSB, MMP9 and TRPV2 were able to significantly predict disease recurrence. Moreover, tumor grade was also able to significantly predict disease recurrence (Supplementary Table 6). As such, ROC curves showed that TRPV2 and tumor grade were the best predictors for recurrence in this data set, with AUC values of 0.707 and 0.708, respectively (Fig. 7). These parameters were further used in a multivariate logistic regression model to predict disease recurrence (Supplementary Table 5). In this model, both TRPV2 gene expression and tumor grade remained independent predictors for disease recurrence. Altogether, these data suggest TRPV2 gene expression in the primary tumor biopsy as a valuable predictor for disease recurrence.

### TRP channel expression in primary endometrial cancer cells of variable EMT status

The gene expression data suggest that the expression of specific TRP channels correlates with the EMT status of the tumor in both primary biopsies and metastatic biopsies. However, tumor biopsies comprise two distinct but interdependent compartments: the neoplastic cancer cells (of epithelial origin) and the surrounding stroma, both contributing to the observed gene expression in our samples.

**Fig. 8:**
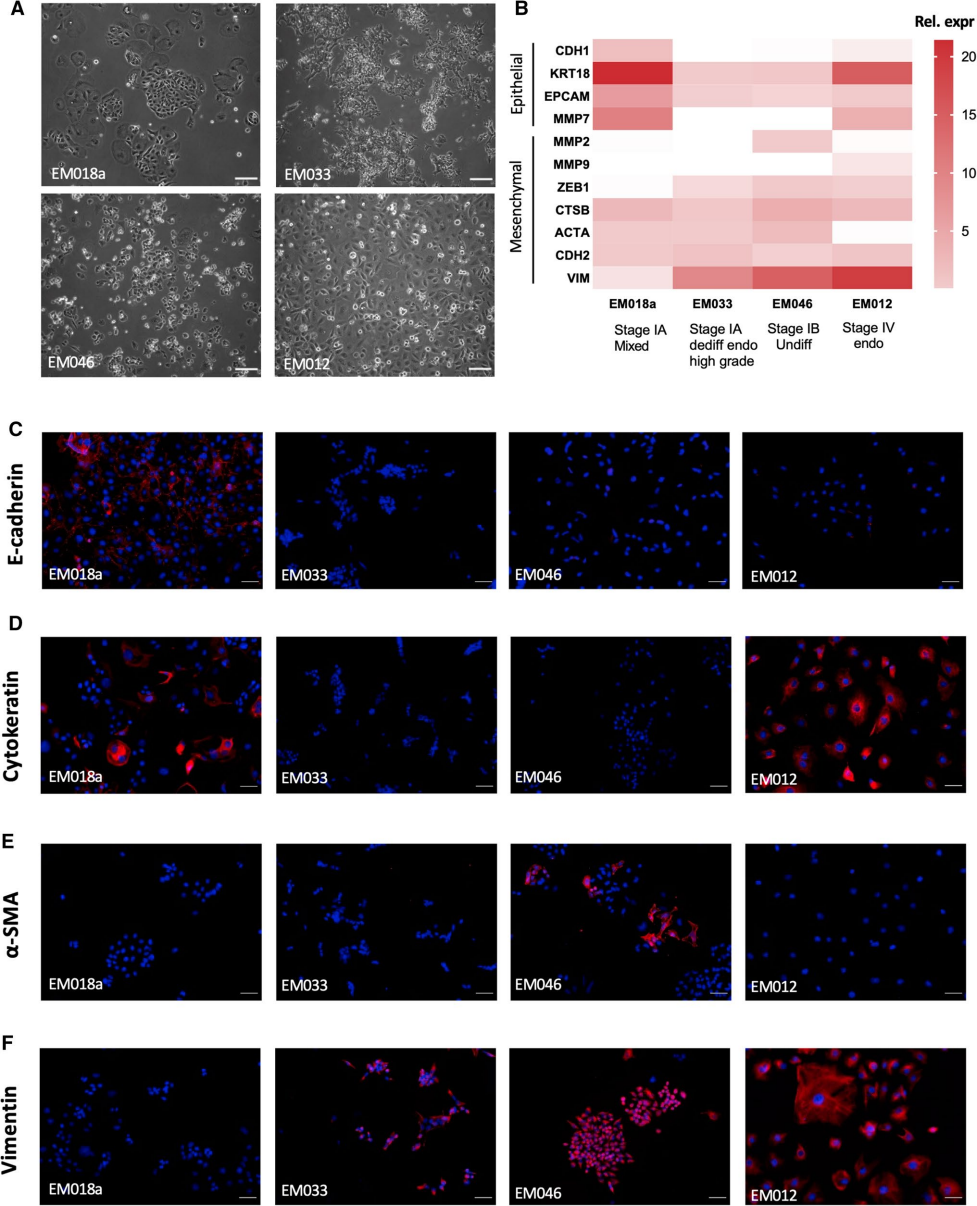
Characterization of EMT status of EC cells: **A** Brightfield pictures (magnification: 10×) of different EC cells EM018a, EM012, EM033, EM046. Scale bar = 100 µm. **B** Heat map of mRNA expression of epithelial (CDH1, KRT18, EPCAM, MMP7) and mesenchymal (MMP2, MMP9, ZEB1, CTSB, ACTA2, CDH2 and VIM) markers genes. Data are represented as relative expression to housekeeping genes HPRT and PGK. Stage and histology of the corresponding cells is indicated on the figure. **C**–**F** Immunofluorescence stainings (magnification: 20×) of the epithelial markers E-cadherin (**C**) and cytokeratin (**D**) and mesenchymal markers MMP2 (**E**) and vimentin (**F**) in EC cells EM018a, EM012, EM033, EM046. Scale bar = 50 µm.

Hence, TRP channel expression was evaluated in primary endometrial epithelial cancer cells, isolated from tumor biopsies with different characteristics regarding tumor stage and histology (Fig. 8A). Validation and characterization of the cells are described elsewhere [17]. However, their EMT status has not yet been determined. M/E ratios based on mRNA expression of epithelial and mesenchymal marker genes were determined for all four cell cultures (Supplementary Table 7). The EM018a cells were derived from a stage IA tumor with mixed histopathology, comprising both endometrioid and serous components. Only the EM018a cells displayed obvious expression of epithelial markers E-cadherin (CDH1) and cytokeratin (KRT), both at the protein and mRNA level (Fig. 8B–D). Moreover, these cells did not display obvious mRNA or protein expression of mesenchymal markers MMP2, α-smooth muscle actin (ACTA2) and vimentin (VIM) (Fig. 8B–F and Supplementary Fig. 5), and displayed a low EMT status based on other marker gene expression (M/E ratio < 1; Supplementary Table 7). The EM033 cells, derived from a stage IA tumor with dedifferentiated endometrioid histology, displayed a high EMT status, characterized by absence of E-cadherin (CDH1) and cytokeratin (KRT) and the presence of vimentin (VIM) on both protein and mRNA level (Fig. 8B–F). However, these cells did not show expression of MMPs or α-smooth muscle actin (ACTA2) (Fig. 8B,  E;  Supplementary Fig. 5). In contrast, EM046 cells, isolated from a stage IB undifferentiated tumor, displayed both MMP2 and α-smooth muscle actin expression, as well as vimentin on both protein and mRNA level. This, together with the lack of epithelial marker expression on both protein and mRNA level, is suggestive for a high EMT status of the cells (Fig. 8B–F; Supplementary Fig. 5). Indeed, of all the four cell lines, EM046 cells display the highest M/E ratio (Supplementary Table 7). Lastly, the EM012 cells were isolated from a stage IV high-grade endometrioid tumor. These cells display a moderate EMT status, with retained expression of cytokeratin both on mRNA and protein level, and MMP7 on mRNA level, but loss of E-cadherin expression. Moreover, some mesenchymal genes are highly expressed, including vimentin (both on protein and mRNA level) and MMP9 (Fig. 8B–F). This moderate EMT status is reflected in a M/E ratio of only 1.04 (Supplementary Table 7).

**Fig. 9:**
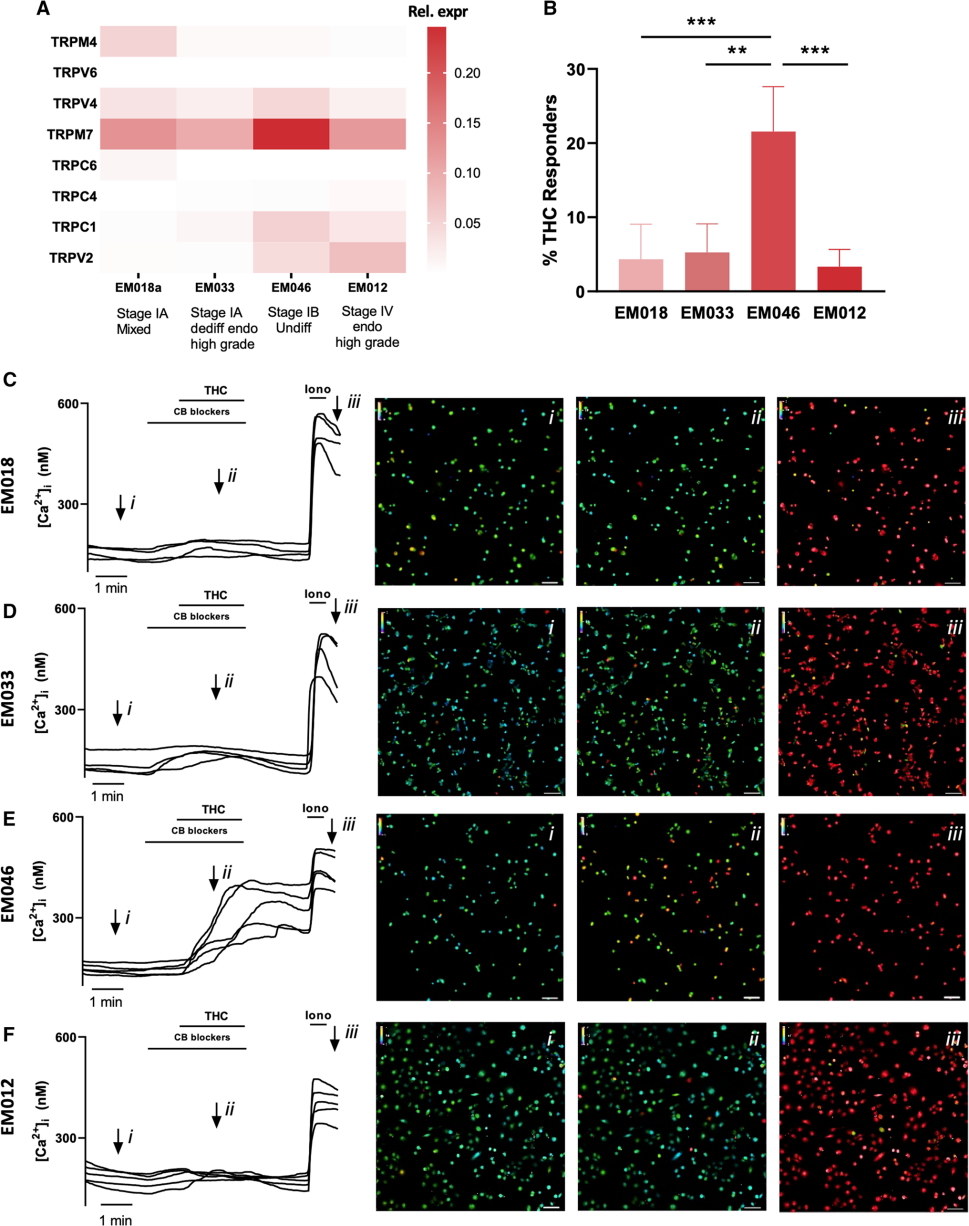
TRP channel expression in EC cells: **A** Heat map of mRNA expression of TRP channels (TRPV2, TRPV4, TRPV6, TRPC1, TRPC4, TRPC6, TRPM4 and TRPM7) in EC cells EM018, EM033, EM046 and EM012. **B** % of THC responders in EM018 (*n* = 504), EM033 (*n* = 678), EM046 (*n* = 718) and EM012 (*n* = 707) cells. **p < 0.01; ***p < 0.001 using One-way ANOVA and Tukey’s multiple comparison test. *N* = 3–5 measurements. Data are represented as mean ± SD. **C**–**F** Representative Ca^2+^ traces of THC-induced [Ca^2+^]_i_ changes in EM018 (**C**), EM033 (**D**), EM046 (**E**) and EM012 (**F**) cells with each line representing a single cell. Representative colour-coded Fura-2 [Ca^2+^]_I_ ratio images during measurement of the cells are indicated on the graphs: basal situation (i), after THC application (ii), and after application of ionomycin (iii). Pseudo-colour ratio images were obtained using Nikon software. Scale bar = 100 µm

Interestingly, in accordance with the results in the tumor biopsies, in which TRPV2 expression was associated with increased tumor stage, TRPV2 mRNA expression was higher (tenfold increase) in stage IB (EM046 cells) and stage IV (EM012 cells) samples, compared to the stage IA cell lines EM018a and EM033 (Fig. 9A). Similarly, TRPC1 mRNA expression was the highest in stage IB and stage IV cells, moderate in EM033 cells that underwent EMT and low in EM018 cells with a low EMT status. In contrast, TRPM4 mRNA expression was high in the EM018 cell line (stage IA with CDH1 expression intact), but undetectable in other cell lines. The TRPC4, TRPC6 and TRPV6 expression levels were low in all cells, while TRPM7 was highly expressed in all different cell cultures, with a twofold higher expression in EM046 cell types compared to the other cell lines (Fig. 9A). Overall, these results suggest that, like in tissue biopsies, the expression of TRPV2 and TRPC1 are associated with increased EMT status (or invasiveness), while TRPM4 expression is correlated with a low EMT status in endometrial cancer cells.

From all previous results, TRPV2 mRNA expression emerges as a strong biomarker for progressive EC, both in whole tumor biopsies and EC cells. The functional presence of the channel at the plasma membrane of the cancer cells would provide a strong indication that the channel might be involved in cellular processes driving cancer progression. Therefore, the functional expression of TRPV2 was investigated using calcium microfluorimetry. Stimulation of EC cells EM018a, EM033, EM046 and EM012 by THC (50 µM), including pre- and co-application of CB receptor blockers, induced a robust calcium influx only in the EM046 population (Fig. 9B–F). Indeed, while the other EC cell populations could not reach a responder percentage above 5%, a significantly higher amount of (21.5 ± 6.0%) of the EM046 cells responded to THC application, which suggest the expression of functional TRPV2 at the plasma membrane in these EC cells with a high EMT status (Fig. 9B).

## Discussion

Functional expression of specific TRP channels has been associated with cellular invasion, migration, and differentiation in response to extracellular cues. Dysregulation of signaling pathways in pathological conditions such as cancer can modulate the expression level of certain TRP channels, thereby altering cell sensitivity and response options to the extracellular environment. To date, TRP channel transcription signatures have never been linked to epithelial and mesenchymal phenotypes, and the association between these signatures and endometrial cancer development is very limited. In this study, evidence is provided for the correlation between the mRNA expression of specific TRP channels and epithelial or mesenchymal cell phenotypes. As such, TRPV2 and TRPC1 are associated with the mesenchymal cell phenotype and are upregulated in both endometrial cancer biopsies and cancer cells with a higher EMT status. In contrast, TRPM4 is linked with an epithelial phenotype and its mRNA expression is higher in cancer biopsies and cancer cells with a lower EMT status.

### TRPV2 mRNA expression as a marker for tumor invasion

TRPV2 is expressed in various tissues, including lung, pancreas and placenta [23, 24]. TRPV2 activity can be modulated by physical modulators like mechanical stimulation. Interestingly, the channel can be activated by the cannabinoid ∆9-tetrahydrocannabinol (THC) and modulated by growth factor signaling [25]. Indeed, TRPV2 was first identified as being a growth factor-regulated channel as it mediated IGF-1 induced Ca^2+^ entry in breast cancer cells [26]. So far, the exact physiological role of TRPV2 remains an enigma, since pharmacological tools to investigate the channels’ function are rather scarce. Nevertheless, a role for TRPV2 in cellular motility has been described by various studies [27, 28]. Here, we demonstrated that functional expression of TRPV2 is linked to the mesenchymal phenotype in the endometrium, since induction of decidualization (MET) drastically reduced functional TRPV2 expression in ESC, while EMT significantly increased functional TRPV2 expression in EEC. Additionally, our results demonstrated significant correlations between TRPV2 gene expression and several mesenchymal markers in primary and metastatic tumor biopsies, suggesting the channels’ association with the mesenchymal cell phenotype. Moreover, these results illustrated that progression of EC, represented by myometrial invasion status and FIGO stage, coincided with increased TRPV2 mRNA expression in primary tumor biopsies. Additionally, TRPV2 mRNA was identified as an independent predictor for disease recurrence and its expression was significantly increased in metastatic biopsies with a higher EMT status compared to primary biopsies. Furthermore, TRPV2 mRNA expression is associated with detection of MMP mRNA expression in EC cell lines derived from advanced-stage tumors. Remarkably, functional TRPV2 expression was only observed in EM046 cells, the only cell line with validated for MMP2 expression at mRNA and protein level. Moreover, EM046 cells was the sole EC cell line expressing α-smooth muscle actin, thereby confirming the highest EMT status of all investigated EC cell lines. Indeed, protein expression of MMP2 and α-smooth muscle actin is very indicative for EMT, as their expression is also observed in typical mesenchymal ESC cells (Supplementary Fig. 5C, D). In contrast, EM012 cells, which are derived from a stage IV tumor and are expressing high levels of TRPV2 mRNA, do not express both mesenchymal marker proteins. This, together with an intact expression of the epithelial marker protein cytokeratin, suggests that these cells are only in a partial EMT status. Interestingly, these cells showed no response to THC application, suggesting that TRPV2 is not functionally present at the plasma membrane. These results might be indicative for a strong association between functional TRPV2 expression and an invasive cell phenotype, characterized by MMP2 protein expression. Interestingly, it has been shown that TRPV2 expression and functionality in prostate cancer cells increases the expression of MMPs and CTSB. Moreover, knockdown of TRPV2 results in downregulation of MMP2, MMP9 and CSTB mRNA expression [29]. Furthermore, a role for Ca^2+^ signaling in induction of MMP gene expression has been described in oral squamous cell carcinoma, suggesting that Ca^2+^ influx via opening of TRPV2 might indeed modulate expression of MMPs and cellular invasion [30].

Our results are in line with a recent report regarding the role of TRPV2 in EC, reporting a positive correlation between elevated TRPV2 expression in tumor biopsies, increased tumor stage and shorter progression free survival [31]. Subsequently, increased TRPV2 expression was also observed in other malignancies like breast cancer, esophageal squamous cell carcinoma, gastric cancer, urothelial cancer and prostate cancer [29, 32–35]. Most studies conclude that increased TRPV2 expression and subsequent increased Ca^2+^ influx in cancer cells enhances their migratory capacity and facilitates disease progression. However, it should be considered that tumors are a complex mixture of various cell types, including the neoplastic cancer cells of epithelial origin, and mesenchymal-derived non-malignant cells such as cancer associated fibroblasts and immune cells infiltrating the tumor. Since we demonstrated that TRPV2 is associated with the mesenchymal cell phenotype, the potential contribution of mesenchymal tumor components should not be disregarded when evaluating the role of TRPV2 in cancer. Furthermore, these mesenchymal components often promote tumor progression themselves [36], further complicating the interpretation of TRPV2 mRNA expression in tumor biopsies. Therefore, the biological role of TRPV2 in non-malignant cell types within the tumor microenvironment should be examined alongside ectopic TRPV2 expression within malignant cells. Unfortunately, most studies investigating the expression of TRPV2 in cancer only focus on tumor cells, possibly overlooking an important contribution of the channel in the tumor microenvironment. In our study, TRPV2 expression showed no clear association with tumor cell specific clinicopathological characteristics such as tumor grade, histological and molecular classification, suggesting that examining merely the neoplastic tumor compartment might be insufficient to explain its expression profile. Nevertheless, our results obtained in EC cells suggest that increased functional expression of TRPV2 in cancer cells might contribute, at least in part, to endometrial cancer progression.

### TRPC1 expression correlates with tumor EMT status

Ca^2+^ influx via TRPC1 is known to contribute towards Ca^2+^-mediated gene expression important for cellular migration, proliferation, and survival. Our results indicate that similar to TRPV2, also TRPC1 mRNA expression is associated with the mesenchymal phenotype of the endometrium. The channel is moderately expressed in ESC, and significantly downregulated upon induction of MET. In contrast, a robust upregulation of TRPC1 mRNA expression could be observed after induction of EMT in EEC, albeit not significantly. Moreover, TRPC1 mRNA expression strongly correlates with mesenchymal gene expression in both primary and metastatic biopsies. In primary biopsies, TRPC1 expression correlates with high tumor grade, and is significantly higher in serous and carcinosarcoma tumors compared to endometrioid tumors. Interestingly, increased TRPC1 expression was observed in tumors that were categorized in the p53 abnormal molecular subclass, which is associated with worse disease prognosis. Additionally, TRPC1 expression is significantly increased in metastatic tumor biopsies compared to primary biopsies. Contrary to TRPV2, TRPC1 mRNA expression does show a strong association with tumor cell specific clinicopathological characteristics, which is reflected in the results obtained using EC cells. While TRPV2 mRNA expression is mainly correlated to stage and invasion status of the cell, i.e., expression of MMPs, TRPC1 mRNA expression is increased in all cell lines that underwent EMT, regardless the tumor stage or MMP expression.

These data are in line with earlier observations regarding TRPC1 expression in other cancer types, including pancreatic, breast, lung and colon cancer and glioblastoma [37–46]. Studies conducted in breast cancer tissue revealed that the claudin-low breast cancer subtype exhibited the highest TRPC1 expression levels compared to other subtypes. Moreover, in triple negative breast cancer, the mesenchymal subtype showed the highest expression level of TRPC1, while the basal subtype with lymph-node metastasis was associated with high TRPC1 expression and worse prognosis [47]. In vitro studies describe the importance of TRPC1-mediated Ca^2+^ influx in proliferation and invasion of cancer cells. Indeed, knockdown of TRPC1 expression or pharmacological inhibition of TRPC1 and/or SOCE activity decreased cytosolic Ca^2+^ levels and abrogated cancer cell proliferation and motility [37]. Interestingly, some of these studies report increased TRPC1 expression after hypoxia-induced EMT via HIF-1α  signaling in cancer [38, 48]. Furthermore, reducing TRPC1 expression inhibited hypoxia-induced increased Snail, Vimentin, and Twist expression. Our data indicated that gene expression of ZEB1, a major transcription factor in EMT, can predict TRPC1 mRNA expression, both in primary and metastatic tumors. These results suggest an important role of TRPC1 in EMT and underline the link between TRPC1 expression and the mesenchymal cell phenotype. However, the potential contribution of TRPC1 signaling in the non-malignant mesenchymal compartment of the tumor should not be underestimated when investigating the exact role of TRPC1 in cancer progression.

### TRPM4 expression as a hallmark for low-risk tumors

Dysregulation of TRPM4 expression and functioning has been described in several diseases, including cancer [49–54]. In contrast to other TRP channels, TRPM4 is a Ca^2+^ activated monovalent cation channel. Activation of the channel by increased levels of cytosolic Ca^2+^ induces influx of cations, which causes membrane depolarization and reduces the driving force for Ca^2+^ entry via other ion channels, including store-operated calcium channels [55]. Indeed, it was shown that Na^+^ influx via TRPM4 results in a decreased Ca^2+^ influx in many different cell types, including various immune and cancer cells [49, 56–58]. Our results showing high expression of TRPM4 in EEC are in line with earlier reports, illustrating high TRPM4 expression in both human and mouse EEC [18, 59]. Interestingly, expression of TRPM4 was significantly decreased after EMT induction, suggesting a strong association with the epithelial cell phenotype. In line herewith, our results showed increased TRPM4 mRNA expression in epithelial-like cancer cell phenotypes, represented by high cellular differentiation in low-grade tumors, and endometrioid histology. TRPM4 mRNA expression was markedly decreased in non-endometrioid and high-grade tumors with a higher EMT status. These findings were confirmed at the cellular level, since TRPM4 expression was only observed in a single EC cell culture in which the CDH1 (E-cadherin) expression was intact.

Our findings are in line with a recently published study regarding TRPM4 expression in EC, which illustrated an association between decreased expression of TRPM4 and unfavorable prognosis and aggressive progression of EC [60]. The authors provided evidence for decreased expression of TRPM4 in tumors with advanced tumor stage, high grade, myometrial invasion, and lymph node metastases. Moreover, they proposed TRPM4 as an independent prognostic variable for overall survival. In the current study, our data illustrated a significant association between TRPM4 mRNA expression and tumor grade. Possibly, our sample size was too limited to detect significant associations between TRPM4 expression and the other clinical parameters listed above. Nevertheless, the overall conclusion that decreased TRPM4 expression is associated with an unfavorable prognosis of EC, corroborates our findings. Interestingly, in this study evidence was obtained that TRPM4 is a negative regulator of EMT in EC cell lines since silencing TRPM4 reduced E-cadherin (CDH1) and cytokeratin (KRT18) protein expression and increased the expression of the mesenchymal marker proteins N-cadherin (CDH2) and vimentin (VIM). These results are in line with our results obtained in EC cells, where TRPM4 mRNA expression is only detected in cells with a low EMT status. Furthermore, it has been suggested that the regulatory effect of TRPM4 on EMT is mediated via p53 signaling [60]. Interestingly, our data identified a significantly reduced expression of TRPM4 in the p53 abnormal molecular subclass compared to the p53 wild-type subclass, suggesting that TRPM4 expression could be linked to p53 signaling in EC.

TRPM4 expression has also been reported as being significantly downregulated in colorectal cancer compared to normal tissue, suggesting a correlation between decreased TRPM4 expression, loss of the epithelial cell phenotype and increased malignant characteristics in carcinomas in general [61]. However, elevated levels of TRPM4 expression have been observed in breast and prostate cancer [50, 62], indicating that its role might be cancer type specific.

### Other TRP channels

TRPM7 is a ubiquitously expressed non-selective cation channel, which includes a C-terminal kinase capable of phosphorylating downstream substrates. TRPM7 plays an important role in Mg^2+^ homeostasis of various cell types. It is highly expressed in both EC biopsies and cell lines and was significantly associated with the steroid responsiveness of tumors and with their metastatic nature. Even though we did not find any other pathological parameter to be associated with TRPM7 mRNA expression alterations in primary tumors biopsies, the channel might have a role in tumor metastasis. Indeed, several reports have suggested a role for TRPM7 in cancer proliferation, migration, and invasion [63–65]. Furthermore, a more recent study described a role for TRPM7 in EMT induction via PI3K signaling in ovarian cancer cells [66]. However, TRPM7 is considered as a housekeeping TRP channel as it is highly expressed in almost every cell type [67]. Moreover, its expression in ECS and EEC was not altered after phenotypic transitions, and showed little variation when comparing tumor stage, grade, histology, myometrial invasion, molecular subclass in primary tumors and different EC cell lines. Therefore, it is not yet clear whether TRPM7 plays a major role in the progression of EC.

In conclusion, our explorative study is the first to associate TRP channel mRNA expression patterns in healthy epithelial and mesenchymal phenotypes to TRP channel signatures observed in malignant tissues and cells. Notwithstanding the limited sample size (*n* = 54) and median follow-up time (36 months), we were able to observe significant associations between the mRNA expression of TRP channels and clinical parameters and confirm earlier findings regarding TRP channels and (EC) tumor progression. We propose that the TRP channel transcription signature observed in endometrial epithelial and mesenchymal cells can serve as a roadmap to identify high risk EC. Especially TRPV2 and TRPC1 mRNA expression could serve as potential biomarkers for tumor invasiveness. Moreover, we suspect that these findings can be extrapolated to other cancer types, since both channels have been linked to cancer progression in other types of cancer. However, since this study is mainly focused on TRP channel mRNA expression, the exact functional role of these TRP channels in both healthy endometrial cells and EC progression is still unspecified, and requires further investigation.

The potential use of TRP channels as therapeutic targets in cancer has been proposed by several authors [68, 69]. Indeed, altered expression patterns in cancer together with a readily available presence of TRP channels at the cell surface make them interesting targets for larger molecules, such as antibody-based drugs, as well as pharmacological modulators. However, the relatively broad expression pattern of several TRP channels remains an important factor to consider and is currently the main limitation for including TRP channel targeted therapies in cancer treatment.

### Supplementary Information

Below is the link to the electronic supplementary material.

Supplementary file1 (PDF 1030 KB)

## Data Availability

Not applicable.
