# Supplementary material for: TRP channel expression correlates with the epithelial–mesenchymal transition and high-risk endometrial carcinoma
Source: Cell Mol Life Sci. 2021 Dec 22;79(1):26. doi: 10.1007/s00018-021-04023-1 (PMC8732886; doi:10.1007/s00018-021-04023-1)
Supplement: Supplementary file 1 — Supplementary file1 (PDF 1030 KB) [file 18_2021_4023_MOESM1_ESM.pdf]

Supplementary tables and figures

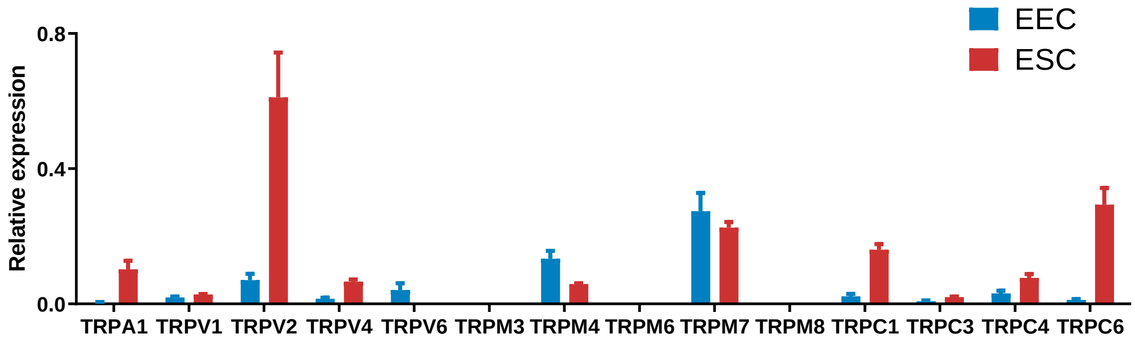

Supplementary figure 1: Quantitative RT-PCR showing TRP channel mRNA expression (TRPA1, TRPV1, TRPV2, TRPV4, TRPV6, TRPM3, TRPM4, TRPM6, TRPM7, TRPM8, TRPC1, TRPC3, TRPC4 and TRPC6, in EEC and ESC. Results are shown as mean ± SEM relative expression to the geometric mean of housekeeping genes HPRT and PGK (published). N=5 p.s. Data used from own earlier publications[1, 2].

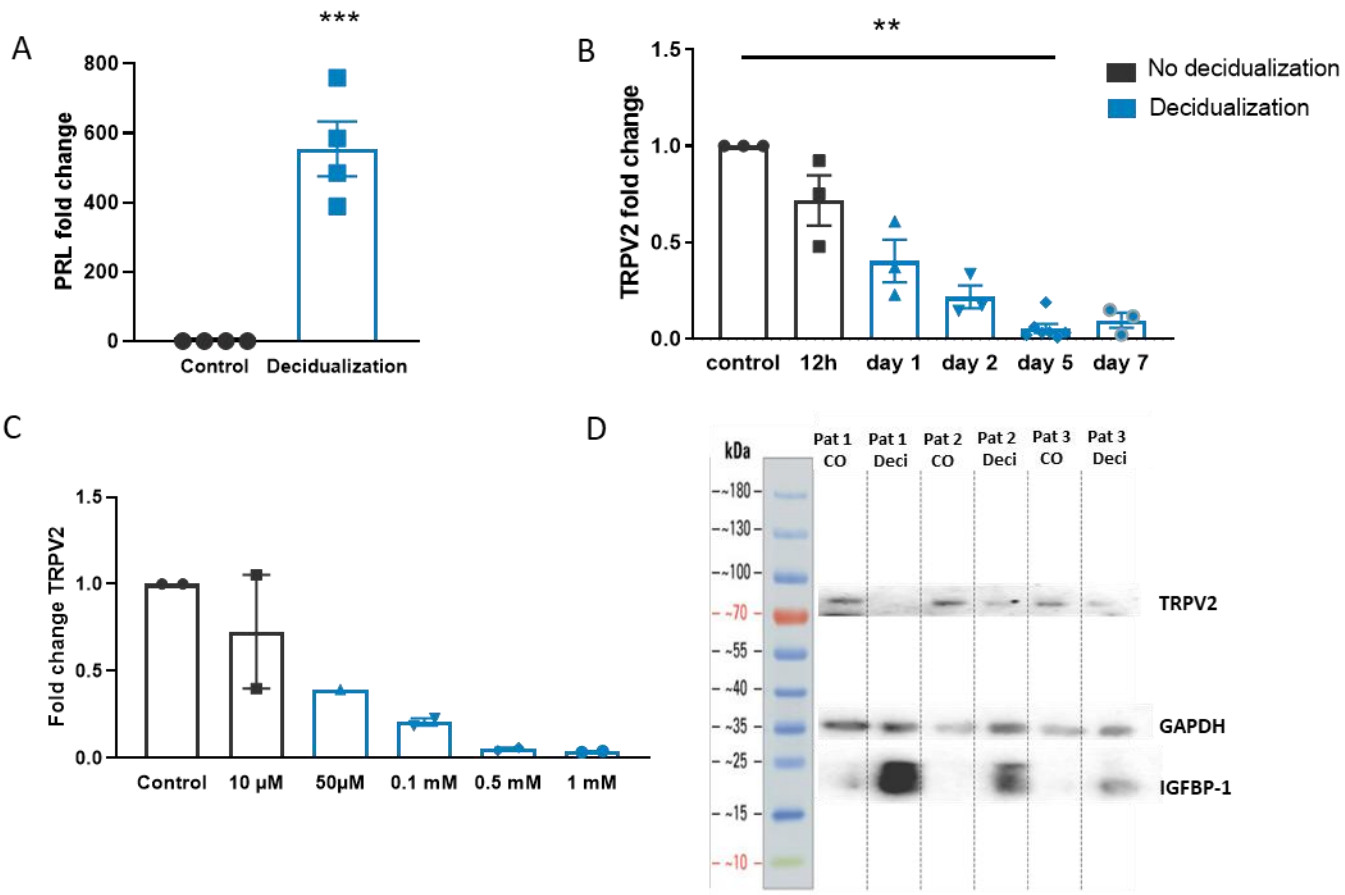

Supplementary figure 2: TRP channels in decidualization. A) Validation of *in vitro* decidualization of HESC. Fold change of PRL mRNA expression after decidualization. **** p < 0,0001 using Wilcoxon signed-rank test on ΔCT values. **B)** Fold change of TRPV2 in response to 12h, 1 day, 2 days, 5 days and 7 days of *in vitro* decidualization. ** p < 0,01 using Kruskal-wallis test + Dunn's multiple comparison test on ΔCt values. N=3-7 p.s **C)** Fold change of TRPV2 in response to 10 µM, 50 µM, 0,1 mM, 0,5 mM and 1 mM 8-Br-cAMP. N=1-2 p.s. All data is shown as mean ± SEM. **D)** Western blot displaying downregulation of TRPV2 protein expression after decidualization in ESC. GAPDH was used as loading control and IGFBP-1 upregulation demonstrates successful decidualization.

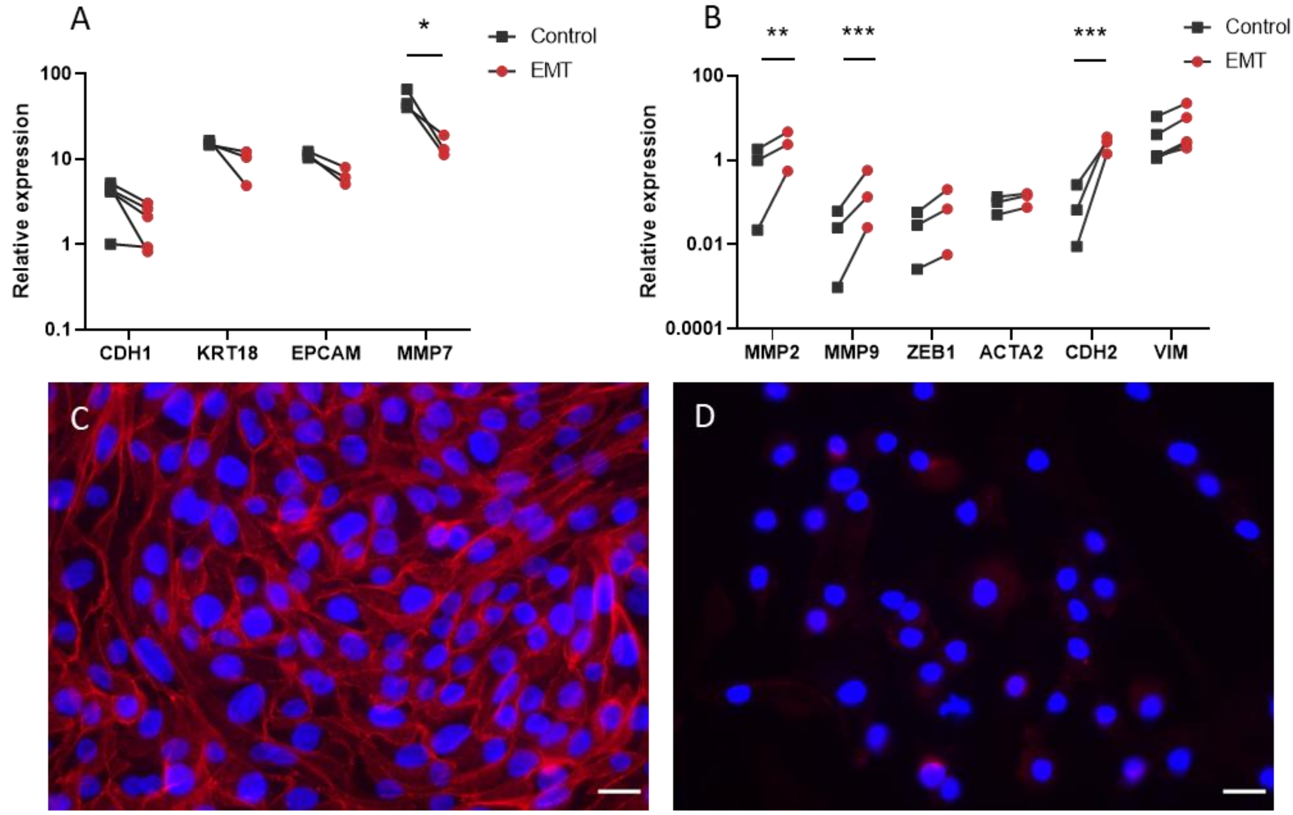

Supplementary figure 3: Validation of *in vitro* EMT induction in EEC. A+B) Quantitative RT-PCR showing mRNA expression of epithelial (CDH1, KRT18, EPCAM, MMP7) and mesenchymal (MMP2, MMP9, ZEB1, ACTA, CDH2, VIM) markers shown as relative expression to housekeeping genes HPRT and PGK. * $p < 0{,}05$, ** $p < 0{,}01$, *** $p < 0{,}001$ using 2-way ANOVA and Sidak's multiple comparison test on ΔCT values. N=3-5 **C+D)** Immunofluorescence staining (magnification: 40x) of the epithelial marker E-cadherin in untreated **(C)** and treated EEC **(D)**. Scalebar = 20 µM

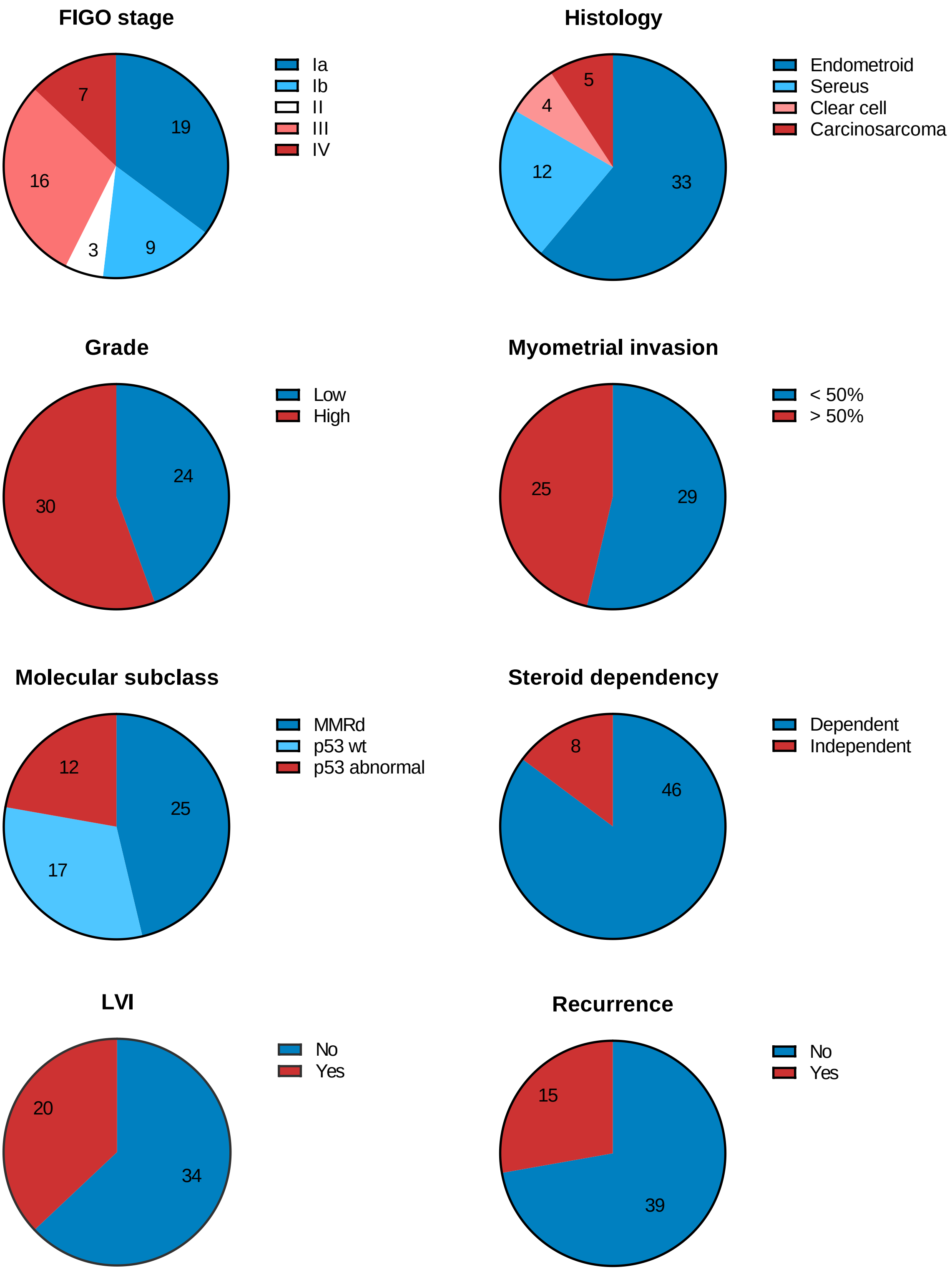

Supplementary figure 4: Patient statistics. A-H) Pie charts displaying number of patients per pathophysiological category: FIGO stage **(A)**, histology **(B)**, grade **(C)**, myometrial invasion **(D)**, molecular subclass **(E)**, steroid dependency **(F)**, LVI **(G)** and recurrence **(H)**.

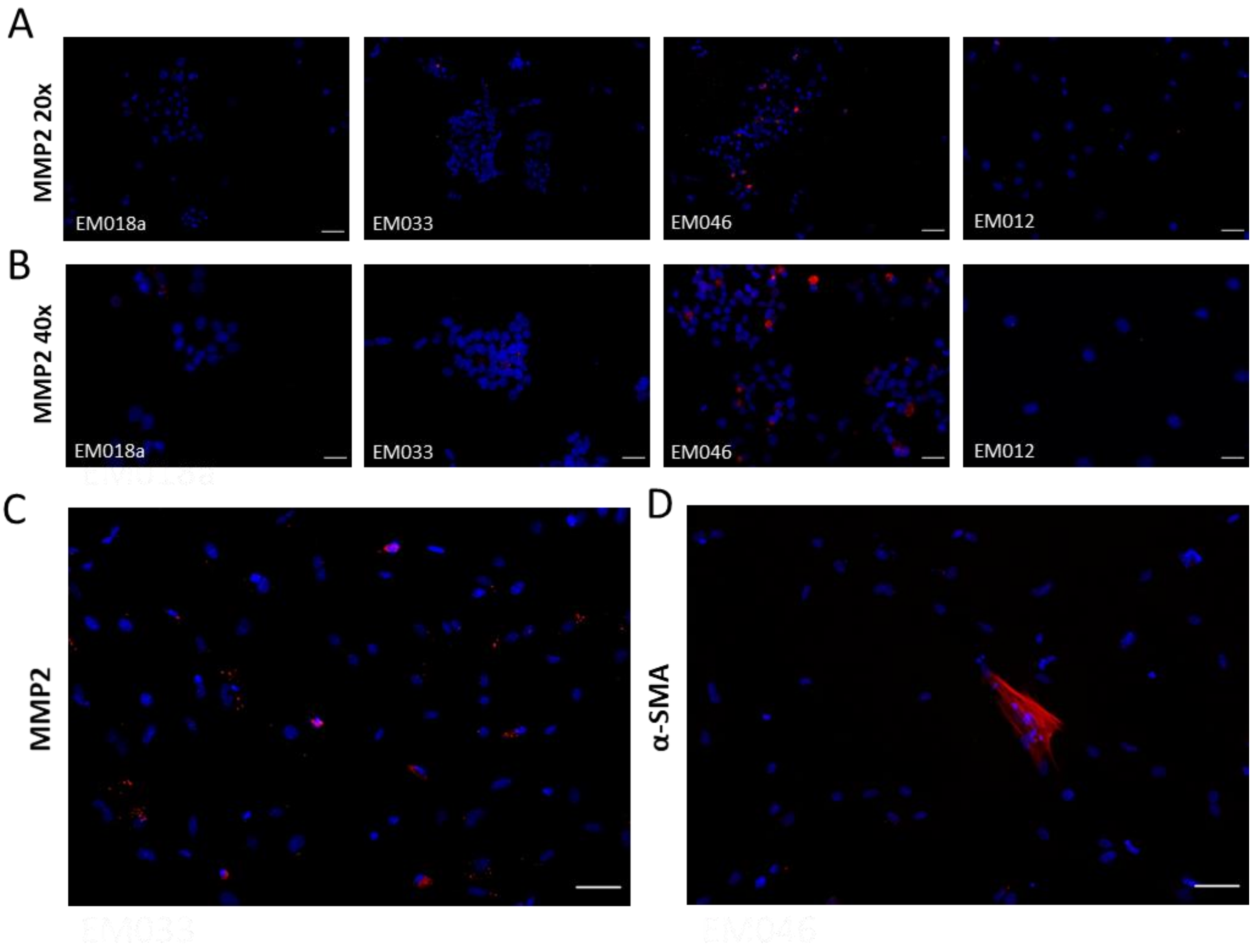

Supplementary figure 5: Characterization of EC cell cultures. A) Immunofluorescence stainings (magnification: 20x) of the mesenchymal marker MMP2 in EM018a, EM012, EM033 and EM046 cells. Scale bar = 50 µm. **B)** Immunofluorescence stainings (magnification: 40x) of the mesenchymal marker MMP2 in EM018a, EM012, EM033 and EM046 cells. Scale bar = 25 µm. **C)** Immunofluorescence staining (magnification: 20x) of the mesenchymal marker MMP2 in ESC cells. Scale bar = 50 µm. **D)** Immunofluorescence staining (magnification: 20x) of the mesenchymal marker α-smooth muscle actin in ESC cells. Scale bar = 50 µm

	R	R Square	Adjusted R Square	Std. Error of the Estimate	Sig.
**TRPV2**	.839	.703	.634	.77015	.000 ***
**TRPV4**	.443	.196	.010	.96833	.419
**TRPV6**	.561	.314	.155	2.27337	.061
**TRPC1**	.844	.713	.646	1.27964	.000 ***
**TRPC4**	.804	.646	.564	1.34367	.000 ***
**TRPC6**	.634	.402	.263	1.49358	.008 **
**TRPM4**	.535	.287	.121	1.11157	.105
**TRPM7**	.632	.399	.259	.66106	.008 **

Supplementary Table 1: SPSS output of multiple linear regression analysis of TRP channel expression (dependent variable) and marker genes (independent variables) in primary biopsies displaying R, R square, adjusted R square, SE and p-values for each TRP channel. . * p < 0,05, ** p < 0,01, *** p < 0,001

		B	SE	t	Sig.
**TRPV2**	CDH1	-.223	.128	-1.748	.080
	EPCAM	.025	.149	.169	.866
	KRT18	.160	.159	1.007	.314
	MMP7	-.006	.047	-.138	.890
	CDH2	-.093	.066	-1.406	.160
	CTSB	.227	.129	1.765	.078
	ACTA2	.043	.093	.463	.643
	MMP2	.233	.105	2.221	.026 *
	MMP9	.231	.049	4.736	.000 ***
	ZEB1	.147	.172	.853	.394
**TRPC1**	CDH1	.069	.212	.326	.744
	EPCAM	.121	.248	.489	.625
	KRT18	-.179	.265	-.677	.498
	MMP7	-.372	.078	-4.771	.000 ***
	CDH2	.094	.109	.855	.393
	CTSB	.659	.214	3.083	.002 **
	ACTA2	-.038	.154	-.248	.804
	MMP2	.032	.175	.186	.853
	MMP9	-.188	.081	-2.324	.020 *
	ZEB1	1.102	.285	3.861	.000 ***

Supplementary Table 2: SPSS output of multiple linear regression analysis of TRP channel expression (dependent variable) and marker genes (independent variables) in primary biopsies displaying B coefficient, SE, t- and p-values for all marker genes in the model. * p < 0,05, ** p < 0,01, *** p < 0,001

	R	R Square	Adjusted R Square	Std. Error of the Estimate	Sig.
**TRPV2**	.931	.867	.810	.72893	.002 **
**TRPV4**	.797	.636	.595	.53922	.003 **
**TRPC1**	.853	.728	.659	1.07810	.006 **
**TRPC4**	.666	.443	.381	1.35545	.025 *
**TRPC6**	.772	.596	.495	.87412	.027 *
**TRPM4**	.623	.388	.320	1.00119	.041 *

Supplementary Table 3: SPSS output of multiple linear regression analysis of TRP channel expression (dependent variable) and significantly correlated marker genes (independent variables) in metastatic biopsies displaying R, R square, adjusted R square, SE and p-values for each TRP channel. * p < 0,05, ** p < 0,01

		B	SE	t	Sig.
**TRPV2**	MMP9	.188	.094	2.001	.085
	ZEB1	.990	.318	3.113	.017 *
	ACTA	-.076	.212	-.355	.733
**TRPV4**	CTSB	.512	.129	3.961	.003 **
**TRPC1**	ZEB1	.902	.367	2.459	.039 *
	MMP2	.253	.183	1.384	.204
**TRPC4**	MMP2	.468	.175	2.676	.025 *
**TRPC6**	MMP2	.246	.140	1.756	.117
	CTSB	.348	.260	1.335	.219
**TRPM4**	EPCAM	.234	.098	2.387	.041 *

Supplementary Table 4: SPSS output of (multiple) linear regression analysis of TRP channel expression (dependent variable) and significantly correlated marker genes (independent variables) in metastatic biopsies displaying B coefficient, SE, t- and p-values for all marker genes in the model. * p < 0,05, ** p < 0,01

	B	SE	Sig.	Exp(B) (OR)
**TRPV2 expression**	-.721	.360	.046 *	.486
**Grade**	2.126	.895	.018 *	8.382

Supplementary Table 5: Logistic regression analysis of TRPV2 expression and tumor grade (predictor variables) and disease recurrence (independent variable) displaying B coefficient, SE, p-values and odds ratio for all predictor variables in the model. * p < 0,05

	B	SE	Sig.	Exp(B) (OR)
**TRPV2 expression**	-.662	.303	.030 *	.516
**CTSB expression**	-.678	.324	.037 *	.508
**MMP9 expression**	-.277	.134	.038 *	.758
**Grade (Low vs High)**	2.047	.831	.014 *	7.747

Supplementary Table 6: Logistic regression analysis of TRPV2, CTSB, MMP9 expression and tumor grade (predictor variables) and disease recurrence (independent variable) displaying B coefficient, SE, p-values and odds ratio for all predictor variables in the model. * p < 0,05

	M/E ratio
**EM018a**	0,5714286
**EM033**	1,1428571
**EM046**	1,7142857
**EM012**	1,047619

Supplementary Table 7: M/E ratio for EC cancer cell cultures

1. Hennes, A., et al., *Functional expression of the mechanosensitive PIEZO1 channel in primary endometrial epithelial cells and endometrial organoids.* Scientific Reports, 2019. **9**.
2. De Clercq, K., et al., *Functional expression of transient receptor potential channels in human endometrial stromal cells during the luteal phase of the menstrual cycle.* Hum Reprod, 2015. **30**(6): p. 1421-36.
